# Hsp90 Governs Dispersion and Drug Resistance of Fungal Biofilms

**DOI:** 10.1371/journal.ppat.1002257

**Published:** 2011-09-08

**Authors:** Nicole Robbins, Priya Uppuluri, Jeniel Nett, Ranjith Rajendran, Gordon Ramage, Jose L. Lopez-Ribot, David Andes, Leah E. Cowen

**Affiliations:** 1 Department of Molecular Genetics, University of Toronto, Toronto, Ontario, Canada; 2 Department of Biology and South Texas Center for Emerging Infectious Diseases, University of Texas at San Antonio, Texas, United States of America; 3 Department of Medicine, University of Wisconsin, Madison, Wisconsin, United States of America; 4 College of Medicine, Veterinary and Life Science, University of Glasgow, Glasgow, United Kingdom; University of Birmingham, United Kingdom

## Abstract

Fungal biofilms are a major cause of human mortality and are recalcitrant to most treatments due to intrinsic drug resistance. These complex communities of multiple cell types form on indwelling medical devices and their eradication often requires surgical removal of infected devices. Here we implicate the molecular chaperone Hsp90 as a key regulator of biofilm dispersion and drug resistance. We previously established that in the leading human fungal pathogen, *Candida albicans*, Hsp90 enables the emergence and maintenance of drug resistance in planktonic conditions by stabilizing the protein phosphatase calcineurin and MAPK Mkc1. Hsp90 also regulates temperature-dependent *C. albicans* morphogenesis through repression of cAMP-PKA signalling. Here we demonstrate that genetic depletion of Hsp90 reduced *C. albicans* biofilm growth and maturation in vitro and impaired dispersal of biofilm cells. Further, compromising Hsp90 function in vitro abrogated resistance of *C. albicans* biofilms to the most widely deployed class of antifungal drugs, the azoles. Depletion of Hsp90 led to reduction of calcineurin and Mkc1 in planktonic but not biofilm conditions, suggesting that Hsp90 regulates drug resistance through different mechanisms in these distinct cellular states. Reduction of Hsp90 levels led to a marked decrease in matrix glucan levels, providing a compelling mechanism through which Hsp90 might regulate biofilm azole resistance. Impairment of Hsp90 function genetically or pharmacologically transformed fluconazole from ineffectual to highly effective in eradicating biofilms in a rat venous catheter infection model. Finally, inhibition of Hsp90 reduced resistance of biofilms of the most lethal mould, *Aspergillus fumigatus,* to the newest class of antifungals to reach the clinic, the echinocandins. Thus, we establish a novel mechanism regulating biofilm drug resistance and dispersion and that targeting Hsp90 provides a much-needed strategy for improving clinical outcome in the treatment of biofilm infections.

## Author Summary

*Candida albicans* and A*spergillus fumigatus* are the most common causative agents of fungal infections worldwide. Both species can form biofilms on host tissues and indwelling medical devices that are highly resistant to antifungal treatment. Here we implicate the molecular chaperone Hsp90 as a key regulator of biofilm dispersion and drug resistance. Compromising Hsp90 function reduced biofilm formation of *C. albicans* in vitro and impaired dispersal of biofilm cells, potentially blocking their capacity to serve as reservoirs for infection. Further, compromise of Hsp90 function abrogated resistance of *C. albicans* biofilms to the most widely deployed class of antifungal, the azoles, both in vitro and in a mammalian model of catheter-associated candidiasis. Key drug resistance regulators were depleted upon reduction of Hsp90 levels in planktonic but not biofilm conditions, suggesting that Hsp90 regulates drug resistance through different mechanisms in these distinct cellular states. Reduction of Hsp90 markedly reduced levels of matrix glucan, a carbohydrate important for *C. albicans* biofilm drug resistance. Inhibition of Hsp90 also reduced resistance of *A. fumigatus* biofilms to the newest class of antifungal, the echinocandins. Thus, targeting Hsp90 provides a promising strategy for the treatment of biofilm infections caused by diverse fungal species.

## Introduction

In recent decades, fungal pathogens have emerged as a predominant cause of human disease, especially in immunocompromised individuals. The number of acquired fungal bloodstream infections has increased by ∼207% in this timeframe [1], [2], [3]. Although diverse species are capable of causing infection, a few prevail as the most prevalent cause of disease. *Candida* and *Aspergillus* species together account for ∼70% of all invasive fungal infections, with *Candida albicans* and *Aspergillus fumigatus* prevailing as the leading causal agents of opportunistic mycoses [2]. *Candida* species are the fourth leading cause of hospital acquired bloodstream infections in the United States with mortality rates estimated at 40% [4], [5]. The profound economic consequences of *Candida* infections can be demonstrated by the ∼$1.7 billion spent annually on treating candidemia in the United States alone [6]. Further, *A. fumigatus* is the most common etiological agent of invasive aspergillosis, with a 40–90% mortality rate [7]. In patients with pulmonary disorders such as asthma or cystic fibrosis, *A. fumigatus* infection can cause allergic bronchopulmonary aspergillosis leading to severe complications. For these fungal species, there are numerous factors that contribute to the pathogenicity and recalcitrance of resulting infections to antifungal treatment, including the ability to evolve and maintain resistance to conventional antifungal therapy [1].

Due to the limited number of drug targets available to exploit in fungal pathogens that are absent or sufficiently divergent in the human host, the vast majority of antifungal drugs in clinical use target ergosterol or its biosynthesis. The azoles are the most widely used class of antifungal in the clinic and function by inhibiting the ergosterol biosynthetic enzyme Erg11, causing a block in the production of ergosterol and the accumulation of the toxic byproduct 14-α-methyl-3,6-diol, culminating in a severe membrane stress [8], [9]. The azoles are generally fungistatic against yeasts, including *Candida* species, and fungicidal against moulds, such as *Aspergillus* species. The fungistatic nature of the azoles towards *C. albicans* culminates in strong directional selection on the surviving population to evolve drug resistance [10], [11]. In fact, high levels of azole resistance in *C. albicans* clinical isolates often accumulate through multiple mechanisms including: upregulation of drug efflux pumps, overexpression or alteration of Erg11, or modification of stress response pathways that are crucial for resistance [1], [10], [11], [12], [13]. The echinocandins are the only new class of antifungal to reach the clinic in decades. They act as non-competitive inhibitors of β-1,3 glucan synthase, an enzyme involved in fungal cell wall synthesis [9], resulting in the loss of cell wall integrity and a severe cell wall stress. The impact of the echinocandins is generally opposite to that of the azoles, in that they are fungicidal against yeasts and fungistatic against moulds. Resistance of *C. albicans* clinical isolates to the echinocandins has been reported and is often associated with mutations in the drug target [13], [14], [15].

An additional key factor responsible for the virulence and drug resistance of *C. albicans* and *A. fumigatus* is their tendency to form biofilms on medical devices that are highly resistant to antifungal treatment [16], [17], [18], [19], [20]. The use of such medical devices — such as venous catheters, urinary catheters and artificial joints — has dramatically risen to more than 10 million recipients per year [21], [22]. This poses a severe clinical problem as *C. albicans* is the third leading cause of intravascular catheter-related infections, and has the overall highest crude mortality rate of ∼30% for device-associated infections [17], [22], [23]. Further, *A. fumigatus* infections have been reported on medical implant devices as well as on bronchial epithelial cells [17], [24]. The inherent drug resistance of biofilms often necessitates surgical removal of the infected medical devices in order to eradicate the fungal infection.

Extensive research has focused on mechanisms of drug resistance in *C. albicans* biofilms, and it is apparent that cells in a fungal biofilm represent an epigenetic modification of the cellular state compared to their planktonic counterparts, with changes in cellular morphology, cell-to-cell communication, and gene expression, as well as with the production of an extra-cellular matrix [16], [18], [20]. Multiple factors contribute to the elevated drug resistance of *C. albicans* biofilms. These factors include increased cell density [25], increased expression of drug efflux pumps [26], [27], decreased ergosterol content [27], elevated β-1,3 glucan levels in the cell wall and biofilm matrix [28], [29], as well as signalling mediated by protein kinase C (PKC) [30] and the protein phosphatase calcineurin [31].

The molecular chaperone Hsp90 regulates complex cellular circuitry in eukaryotes by stabilizing regulators of cellular signalling [32], [33]. As a consequence, inhibiting Hsp90 disrupts a plethora of cellular processes and has broad therapeutic potential against diverse eukaryotic pathogens including the protozoan parasites *Plasmodium falciparum* and *Trypanosoma evansi* as well as numerous fungal species [34], [35], [36]. In the planktonic state, Hsp90 potentiates the emergence and maintenance of resistance to azoles and echinocandins in *C. albicans* at least in part via calcineurin [37]; Hsp90 physically interacts with the catalytic subunit of calcineurin, keeping it stable and poised for activation [38]. Recently, Hsp90 was also shown to enable azole and echinocandin resistance in *C. albicans* via the PKC cell wall integrity pathway [39]. Hsp90 depletion results in the destabilization of the terminal mitogen-activated protein kinase (MAPK) Mkc1, providing the second Hsp90 client protein implicated in drug resistance [39]. Compromising *C. albicans* Hsp90 function renders drug-resistant isolates susceptible in vitro and improves the therapeutic efficacy of antifungals in a *Galleria mellonella* model of *C. albicans* pathogenesis and a murine model of disseminated candidiasis [34]. Compromising *A. fumigatus* Hsp90 also enhances the efficacy of echinocandins both in vitro and in the *G. mellonella* model of infection [34]. Notably, Hsp90 regulates not only drug resistance in *C. albicans* but also the morphogenetic transition between yeast and filamentous growth, a trait important for virulence [40]. Compromising Hsp90 function induces filamentation by relieving Hsp90-mediated repression of cAMP-protein kinase A (PKA) signalling [41]. The ability to transition between morphological states is also critical for biofilm formation and development [42].

Given that Hsp90 governs fungal morphogenesis and drug resistance in planktonic conditions, we sought to investigate if this molecular chaperone also regulates the development and drug resistance of biofilms. We discovered that genetically compromising Hsp90 function reduced but did not block biofilm maturation in vitro and had minimal impact on the ability of *C. albicans* to form robust biofilms in an in vivo rat catheter model,. Genetic depletion of *C. albicans* Hsp90 reduced biofilm dispersal, with the few dispersed cells being largely inviable. Moreover, compromising *C. albicans* Hsp90 function genetically or pharmacologically transformed the azole fluconazole from ineffectual to highly efficacious in eradicating biofilms both in vitro and in a rat catheter model of infection. In stark contrast to planktonic conditions, reduction of *C. albicans* Hsp90 levels genetically in biofilm conditions did not lead to depletion of the client proteins calcineurin or Mkc1, suggesting that Hsp90 regulates drug resistance through distinct mechanisms in these different cellular states. Genetic depletion of Hsp90 reduced glucan levels in the biofilm matrix, providing a compelling mechanism by which Hsp90 might regulate biofilm drug resistance. Finally, in the most lethal mould, *A. fumigatus*, compromising Hsp90 function enhanced the efficacy of azoles and echinocandins in an in vitro model. Our results implicate Hsp90 as a novel regulator of biofilm dispersion and drug resistance, and provide strong support for the utility of Hsp90 inhibitors as a therapeutic strategy for biofilm infections caused by diverse fungal species.

## Results

### Hsp90 is not required for *C. albicans* biofilm formation in vitro or in vivo

**Figure 1:**
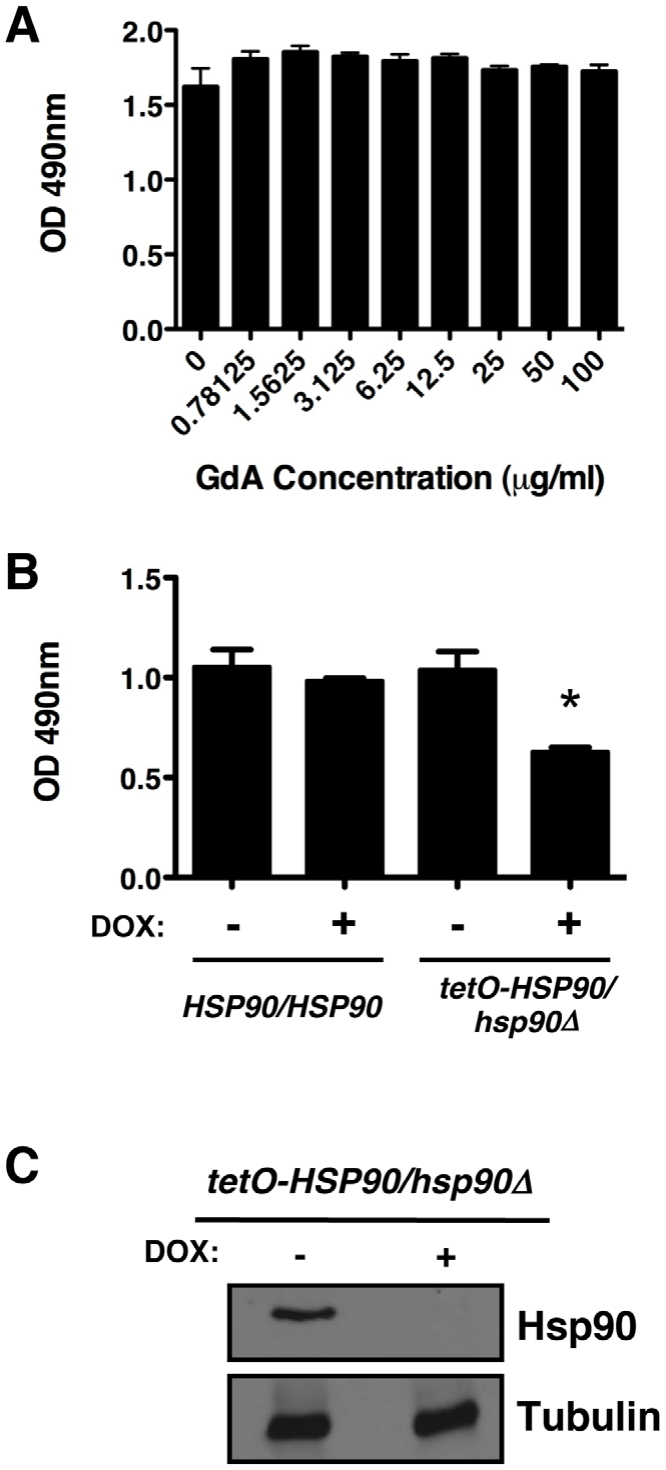
Compromise of Hsp90 function does not block *C. albicans* biofilm development in vitro. (**A**) Biofilms were grown in 96-well microtiter plates in RPMI at 37°C. After 24 hours wells were washed with PBS to remove non-adherent cells and fresh RPMI medium was added containing various concentrations of the Hsp90 inhibitor geldanamycin (GdA). Biofilms were grown for an additional 24 hours at 37°C. Metabolic activity was measured using an XTT reduction assay and quantified by measuring absorbance at 490 nm. Error bars represent standard deviations of five technical replicates. Biofilm growth in the presence of GdA was not significantly different from the untreated control (*P*>0.05, ANOVA, Bonferroni's Multiple Comparison Test). (**B**) Strains of *C. albicans* were grown in 96-well microtiter plates in RPMI at 37°C for 24 hours with or without 20 µg/mL doxycycline (DOX). Metabolic activity was measured as in Figure 1A. Doxycycline-mediated transcriptional repression of *HSP90* in the *tetO-HSP90/hsp90*Δ strain yielded a small reduction in biofilm growth (*P*<0.01). Asterisk indicates *P<*0.01 compared to all other conditions. Error bars represent standard deviations from five technical replicates. (**C**) Hsp90 levels are dramatically reduced in a *C. albicans* biofilm upon treatment of the *tetO*-*HSP90/hsp90*Δ strain with 20 µg/mL doyxcycline in RPMI at 37°C. Total protein was resolved by SDS-PAGE and blots were hybridized with α-Hsp90 and α-tubulin as a loading control.

Due to the key roles of Hsp90 in both morphogenesis and drug resistance under planktonic conditions [37], [41], we hypothesized that Hsp90 might also regulate *C. albicans* biofilm formation and drug resistance. First, we tested whether compromising Hsp90 function affected biofilm growth. To do this, *C. albicans* biofilms were cultured for 24 hours in static 96 well microtiter plates, washed to remove non-adherent cells, grown for an additional 24 hours with various concentrations of the Hsp90 inhibitor geldanamycin, and growth was quantified by metabolic activity using an XTT reduction assay [43]. The geldanamycin was added at 24 hours rather than at the initial time point as is the standard for biofilm drug studies since the initial cells are planktonic and much more susceptible to drugs than their biofilm counterparts [31], [43]; consistent with this, initial attempts to include geldanamycin during inoculation led to a toxicity profile identical to that of planktonic cells (data not shown). When geldanamycin was added at 24 hours, no significant differences in metabolic activity were observed at a variety of concentrations tested up to 100 µg/mL (*P*>0.05, ANOVA, Bonferroni's Multiple Comparison Test, Figure 1A). Thus, Hsp90 inhibitors do not compromise biofilm development.

To further explore Hsp90's role in biofilm formation, we exploited a strain of *C. albicans* in which Hsp90 levels could be depleted by tetracycline-mediated transcriptional repression (*tetO-HSP90/hsp90*Δ). Biofilms of the wild type and *tetO-HSP90/hsp90*Δ strain were cultured in static 96 well microtiter plates with or without 20 µg/mL of the tetracycline analog doxycycline from the time of inoculation. Doxycycline was included at this early point given the time required for transcriptional repression to manifest in depletion of Hsp90, and enabled by the absence of toxicity in planktonic cells. Doxycycline-mediated transcriptional repression of Hsp90 decreased biofilm development, but did not block formation of a mature biofilm (Figure 1B, *P*<0.01). We observed comparable results when biofilms were cultured on silicon elastomer squares, and when biofilm growth was monitored by XTT reduction or by dry weight (Figure S1A and B). To determine if depletion of Hsp90 prior to inoculation had a more profound effect on biofilm formation, we performed a comparable assay but in the presence or absence of doxycycline in the overnight culture. Depletion of Hsp90 prior to inoculation did not further reduce biofilm formation but rather led to a biofilm indistinguishable from the no doxycycline control (Figure S1C). Although Hsp90 is essential, this dose of doxycycline causes reduced growth rate of the *tetO-HSP90/hsp90*Δ strain in planktonic cultures but has little effect on stationary phase cell density [41]. Western blot analysis validated that Hsp90 levels were dramatically reduced in biofilms formed by the *tetO-HSP90/hsp90*Δ strain when cultured in the presence of doxycycline (Figure 1C). We note that when biofilms were formed under shaking conditions, the *tetO-HSP90/hsp90*Δ strain had reduced biofilm growth, which was exacerbated in the presence of doxycycline (Figure S1D). Thus, while Hsp90's impact on biofilm development can vary, under most conditions tested compromising Hsp90 function does not block biofilm formation in vitro.

**Figure 2:**
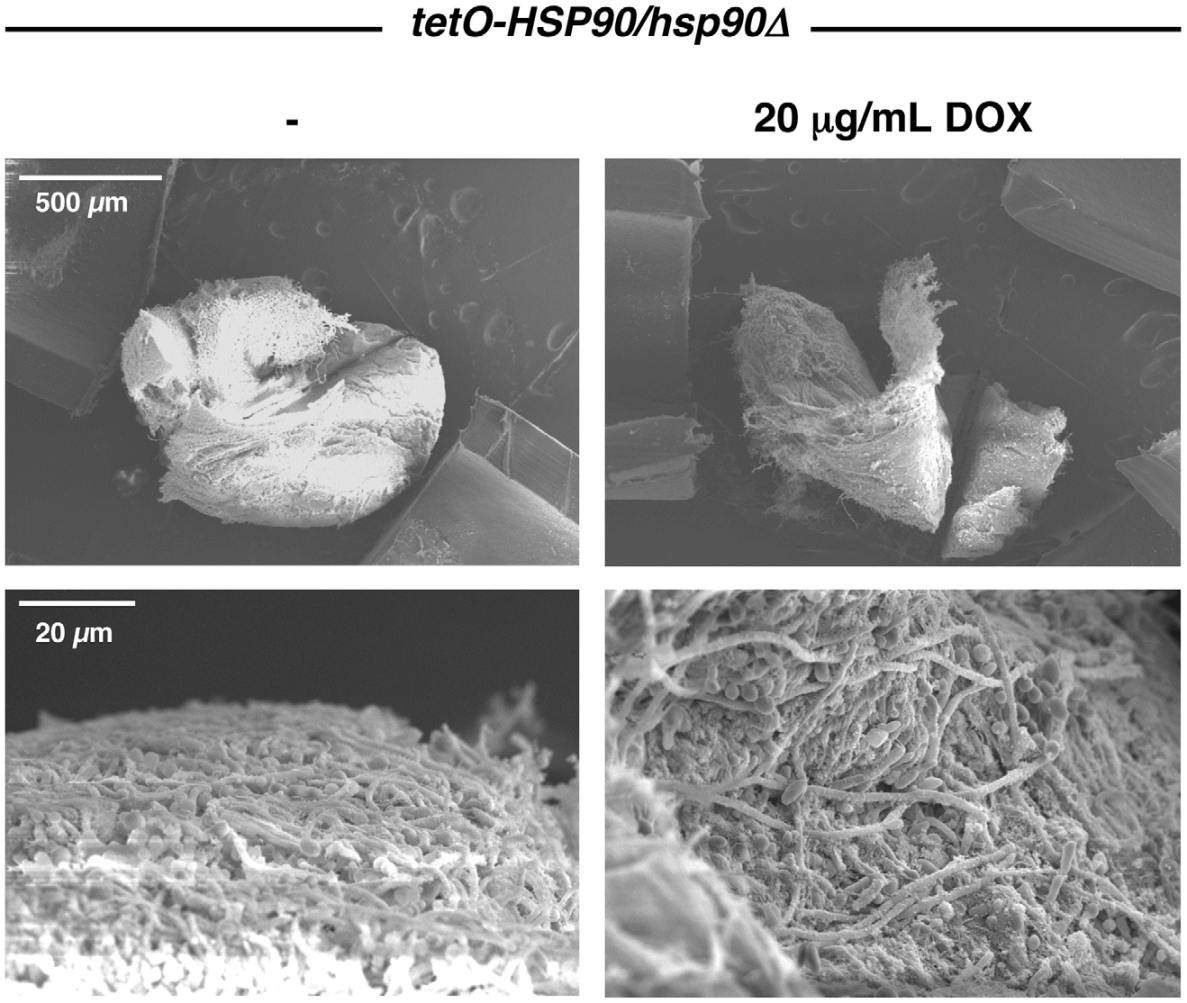
Genetic depletion of Hsp90 does not block *C. albicans* biofilm formation in vivo. The *tetO*-*HSP90/hsp90*Δ strain was inoculated in rat venous catheters in the presence or absence of 20 µg/mL doxycycline (DOX). Biofilms were examined by scanning electron microscopy imaging at 24 hours. The top row represents 50 X magnification while the bottom row represents 1,000 X magnification. Biofilm thickness and structure were similar in the presence or absence of doxycycline.

In order to address the role of Hsp90 in biofilm growth in vivo, biofilm formation was examined using a rat venous catheter model of biofilm-associated candidiasis that mimics central venous catheters in patients [44]. Infection of implanted catheters with *C. albicans* was performed by intraluminal instillation, catheters were flushed after 6 hours, and biofilm formation was monitored with or without 20 µg/mL doxycycline after 24 hours. The *tetO-HSP90/hsp90*Δ strain was capable of establishing a biofilm in the rat venous catheter, as visualized by scanning electron microscopy (Figure 2). Further, transcriptional repression of *HSP90* with doxycycline did not block the formation of a robust biofilm (Figure 2). These results demonstrate that compromising Hsp90 function does not impair the ability of *C. albicans* to form mature biofilms in vivo.

### Compromising Hsp90 function produces biofilms with altered morphologies

**Figure 3:**
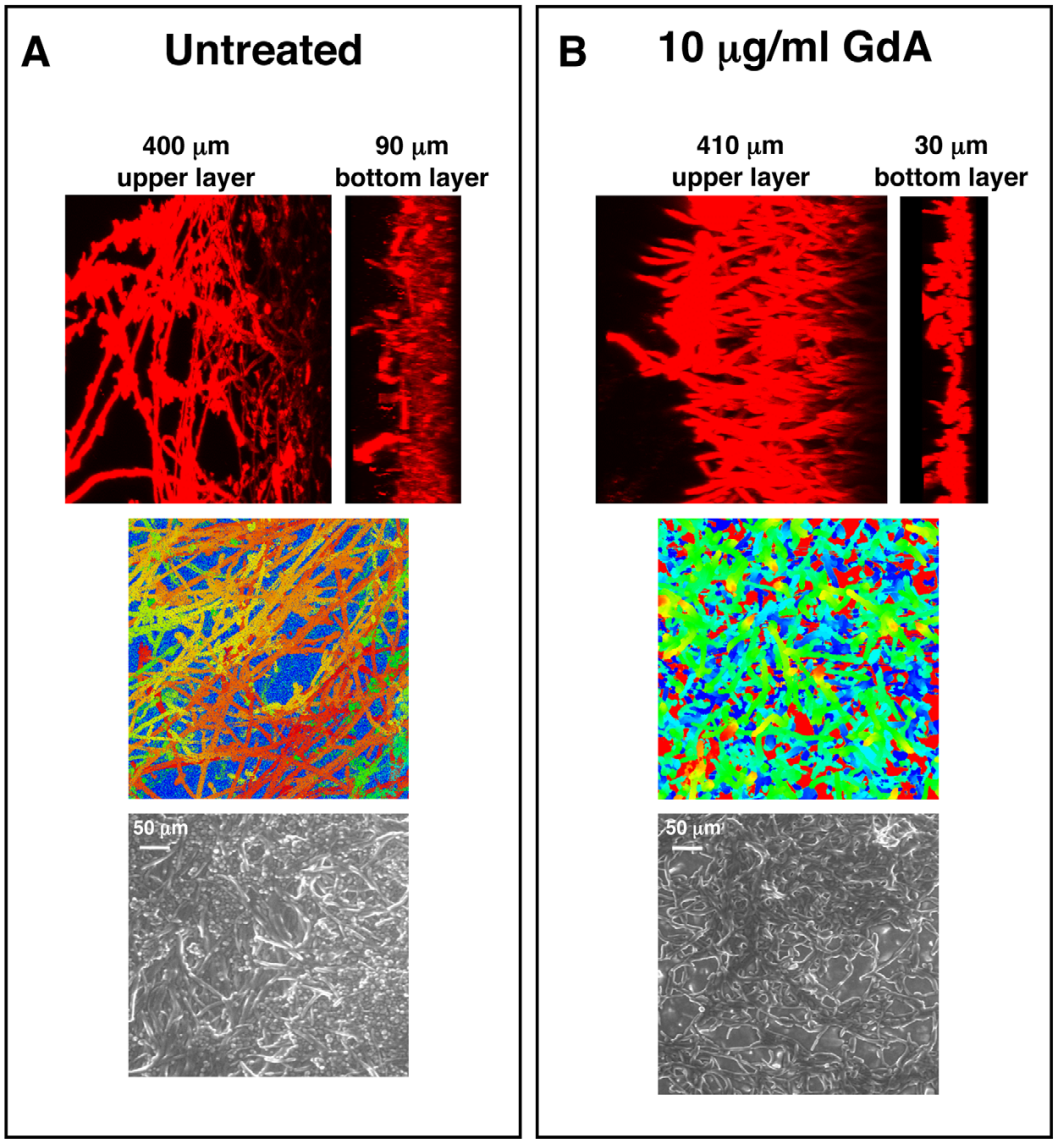
Pharmacological inhibition of Hsp90 alters *C. albicans* biofilms architecture. *C. albicans* cells were grown on silicon elastomer squares in RPMI at 37°C for 24 hours. *C. albicans* wild-type biofilms were left untreated (**A**), or treated with 10 µg/mL geldanamycin (GdA) for 48 hours (**B**). Biofilms were stained with concanavalin A conjugate for confocal scanning laser microscopy visualization, and image reconstructions were created to provide side views (top panel). Representative images are shown. Confocal scanning laser microscopy depth views were artificially coloured (middle panel) with blue representing within 10 µm from the silicon, orange representing approximately 300 µm from the silicon, and red representing over 400 µm from the silicon. Scanning electron microscopy images are shown in bottom panel. Biofilms treated with GdA show a thinner lower layer of yeast than the untreated control.

As mentioned above, Hsp90 is a key regulator of the yeast to filament transition in *C. albicans* [41], a process implicated in virulence and biofilm formation [42]. Therefore, we examined the architecture of geldanamycin treated biofilms cultured on silicon elastomer squares to enable imaging by confocal microscopy. Biofilms treated with geldanamycin had decreased thickness of the bottom yeast layer (30 µm and 45 µm versus 90 µm and 100 µm in the untreated control, *P* = 0.0237, *t*-test) without substantial change in the thickness of the upper layer of filaments (Figure 3). That a greater proportion of the biofilm thickness was occupied by filaments compared to yeast suggests that Hsp90 inhibition might lead to enhanced filamentation in biofilms. Moreover, biofilms treated with geldanamycin showed more polarized filaments extending away from the biofilm basal surface compared to the interconnected meshwork of filaments in an untreated control (Figure 3). That biofilms formed upon Hsp90 inhibition had a greater proportion of their total thickness occupied by filaments compared to yeast is consistent with Hsp90's repressive effect on filamentation in planktonic conditions.

### Hsp90 function is important for dispersal of *C. albicans* biofilms

**Figure 4:**
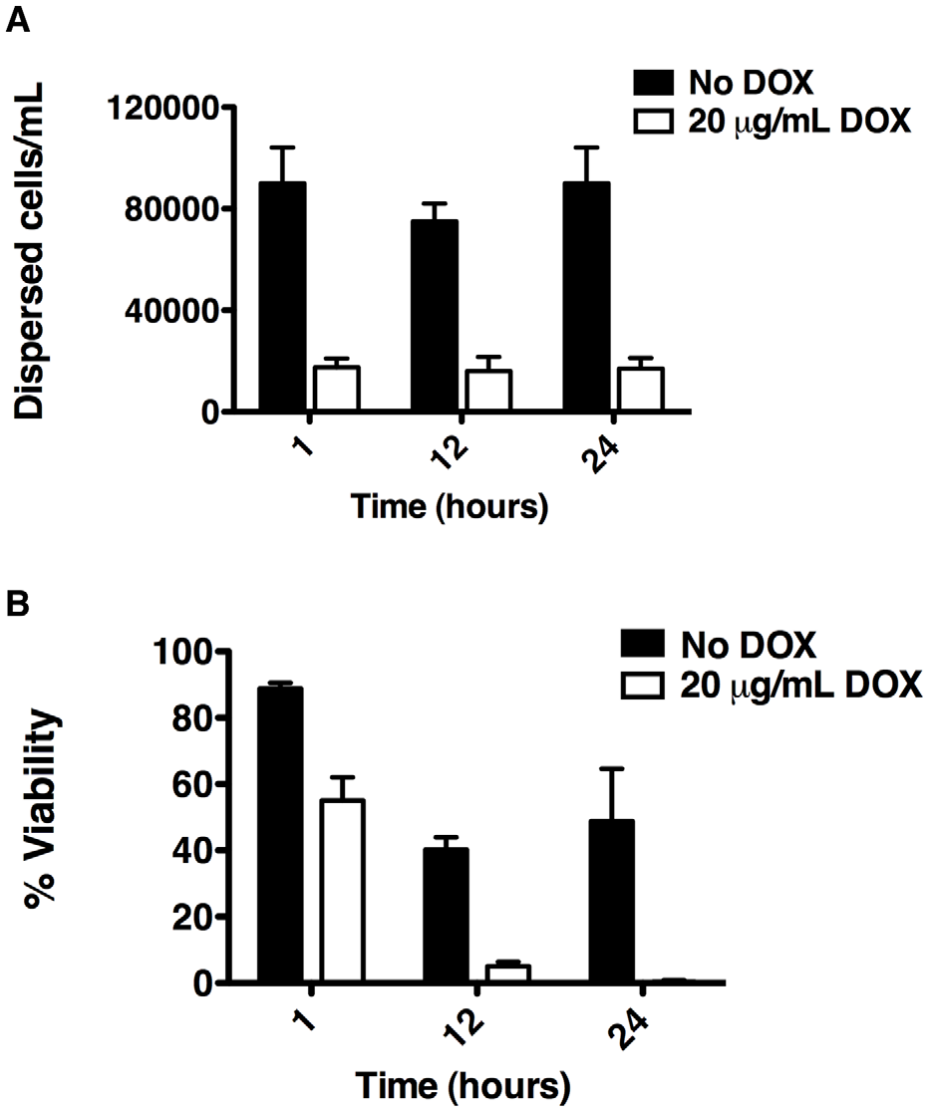
Depletion of Hsp90 reduces biofilm dispersion and viability of the dispersed cell population. *C. albicans* biofilms from the *tetO-HSP90/hsp90Δ* strain were cultured in the presence or absence of 20 µg/mL doxycycline (DOX). (**A**) The number of dispersed cells released from biofilms was monitored over a 24 hour period. (**B**) The viability of dispersed cells was determined by plating on YPD agar.

Based on our finding that *C. albicans* biofilms display altered morphologies upon Hsp90 inhibition, we sought to evaluate the effect of Hsp90 function on biofilm dispersion given that morphogenesis plays a critical role in this process [45], [46]. We monitored dispersion of yeast cells using the only well validated model which involves culturing biofilms on silicon elastomer under conditions of flow [47], [48]. When biofilms were cultured in the absence of doxycycline with the *tetO-HSP90/hsp90*Δ strain, the number of dispersed cells after 1 hour was 90,000 cells/mL and remained fairly constant over a 24 hour time period (Figure 4A). In contrast, in the presence of 20 µg/mL doxycycline the number of dispersed cells was dramatically reduced to approximately 17,000 cells/mL throughout the 24 hours (*P* = 0.0022, *t*-test, Figure 4A). We confirmed that the effects of doxycycline were specifically due to transcriptional repression of *HSP90*, as doxycycline had no impact on biofilm dispersal of the wild-type strain lacking the *tetO* promoter (Figure S2A). Intriguingly, the cells that were dispersed upon reduction of Hsp90 levels had major viability defects compared to their untreated counterparts (*P* = 0.007, *t*-test) with only 55% viable at 1 hour, 5% viable at 12 hours, and less than 1% viable at 24 hours (Figure 4B). The dramatic reduction in viability was specific to the dispersed cell population with doxycycline-mediated transcriptional repression of *HSP90*, as the viability of dispersed cells in the untreated control remained close to 50% even at 24 hours (Figure 4B). Viability was unaffected when a wild-type strain lacking the *tetO* promoter was treated with doxycycline, confirming that the effects observed were due to transcriptional repression of *HSP90* (Figure S2B). The reduced viability upon reduction of Hsp90 levels was specific to the dispersed cell population within the biofilm, as there was only a minor defect in overall metabolic activity of the *tetO-HSP90/hsp90*Δ biofilms in the presence of doxycycline (Figure 1B). Further, under planktonic conditions viability remained>85% when the *tetO-HSP90/hsp90*Δ strain was grown in the presence of doxycycline for 24 hours. Taken together, Hsp90 plays a critical role in the dispersal step of the biofilm life cycle and is crucial for survival of dispersed cells.

### Hsp90 enables the resistance of *C. albicans* biofilms to fluconazole in vitro

**Figure 5:**
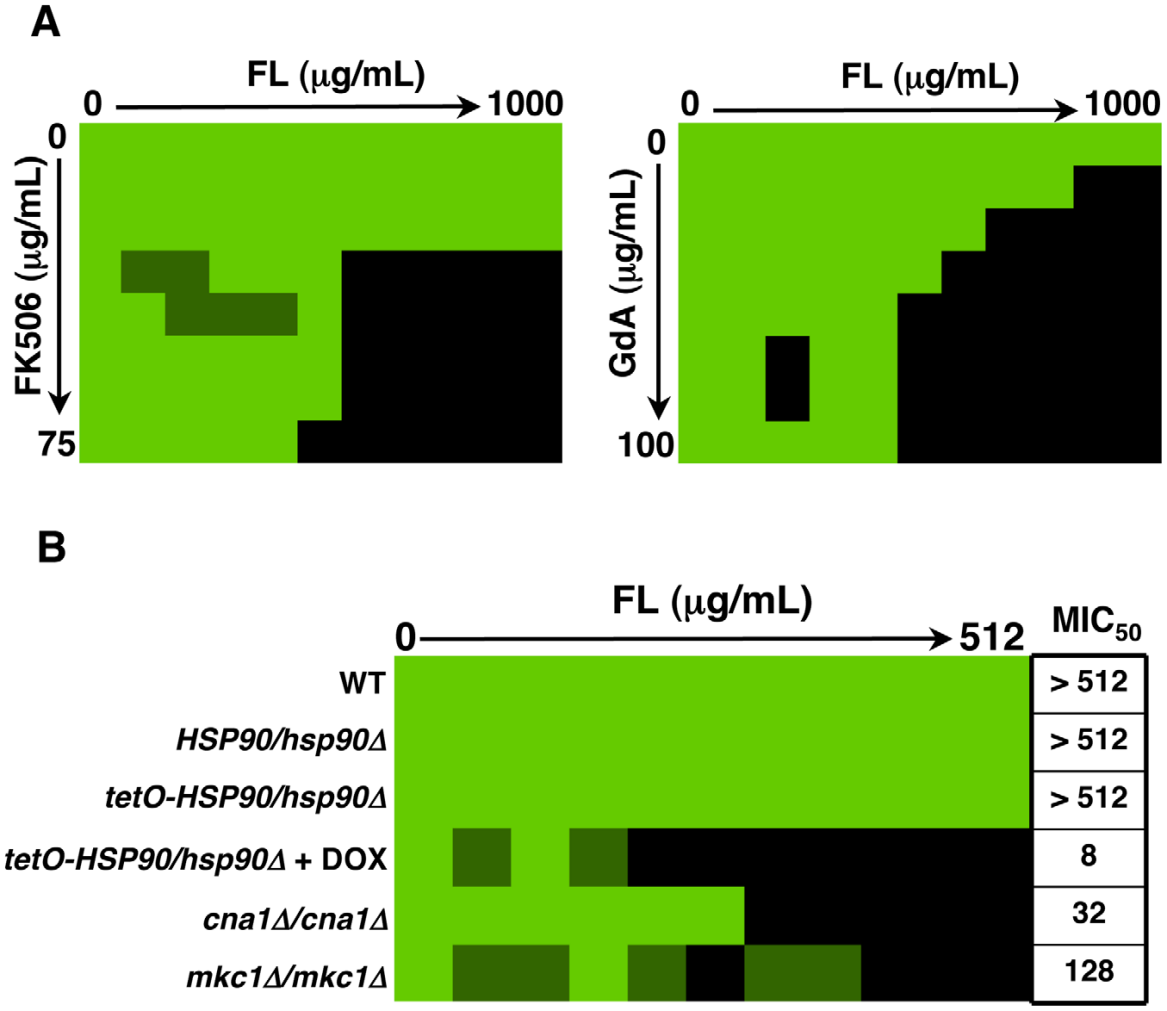
Inhibition of Hsp90 function dramatically enhances the efficacy of fluconazole against *C. albicans* biofilms in vitro. (**A**) Strains of *C. albicans* were grown in 96-well microtiter plates in RPMI at 37°C. After 24 hours cells were washed with PBS to remove non-adherent cells and fresh medium was added with varying concentrations of the azole fluconazole (FL) and either the calcineurin inhibitor FK506 or the Hsp90 inhibitor geldanamycin (GdA) in a checkerboard format. Metabolic activity was measured as in Figure 1A. The FIC index was calculated as indicated in Table 1. Bright green represents growth above the MIC_50_, dull green represents growth at the MIC_50_, and black represents growth below the MIC_50_. Data was quantitatively displayed with colour using the program Java TreeView 1.1.3 (http://jtreeview.sourceforge.net). Inhibiting calcineurin or Hsp90 function has synergistic activity with fluconazole. (**B**) Strains of *C. albicans* were grown in 96-well microtiter plates in RPMI at 37°C. When indicated, 20 µg/mL doxycycline (DOX) was added to the medium. After 24 hours cells were washed with PBS to remove non-adherent cells and fresh medium was added with varying concentrations of fluconazole. Metabolic activity was measured as in Figure 1A. Genetic depletion of Hsp90 reduces the MIC_50_ of fluconazole to a greater extent than deletion of its client proteins calcineurin or Mkc1.

Genetic or pharmacological compromise of Hsp90 function renders *C. albicans* susceptible to azoles and echinocandins under planktonic conditions [37], [38], [49]. Since compromising Hsp90 function pharmacologically did not impair biofilm maturation, we investigated whether inhibition of Hsp90 would alter biofilm drug resistance using the standard 96 well microtiter plate static assay that enables testing many drug concentrations. We focused on the azoles, since biofilms are notoriously resistant to this class of drugs, compromising their therapeutic utility [19]. As a positive control, a wild-type *C. albicans* biofilm was subjected to a gradient of concentrations of the calcineurin inhibitor FK506 in addition to a gradient of fluconazole, a drug combination with established synergistic activity against *C. albicans* biofilms [31]. We confirmed synergistic activity of FK506 with fluconazole by measuring metabolic activity using the XTT reduction assay (Figure 5A). Biofilms were extremely susceptible to the combination of inhibitors with a calculated FIC index of 0.1093, indicating potent synergy (Table 1). To determine if Hsp90 enables biofilm azole resistance, we used an equivalent experiment but with a gradient of concentrations of the Hsp90 inhibitor geldanamycin and a gradient of fluconazole. Geldanamycin exhibited potent synergy with fluconazole, dramatically reducing azole resistance at only 3.125 µg/mL geldanamycin. Maximal effects were observed with 12.5 µg/mL geldanamycin, which reduced the MIC_50_ of fluconazole from >1000 µg/mL to 31.25 µg/mL (Figure 5A). Further, FIC indexes as low as 0.125 to 0.156 were calculated for the combination of fluconazole and geldanamycin confirming that inhibition of Hsp90 has a potent synergistic effect with azoles against *C. albicans* biofilms (Table 1).

**Table 1:** Inhibition of calcineurin or Hsp90 has synergistic activity with fluconazole against wild-type *C. albicans* biofilms.

Inhibitor, concentration range (µg/mL)	Fluconazole concentration range (µg/mL)	FIC index^a^
FK506, 4.6875–75	62.5–1000	0.1093
GdA, 6.25–100	62.5–1000	0.125
GdA, 3.125–100	125–1000	0.156

a FIC index (MIC_50_ of drug A in combination)/(MIC_50_ of drug A alone) + (MIC_50_ of drug B in combination)/(MIC_50_ of drug B alone). A FIC of <0.5 is indicative of synergism.

Next, we utilized the *tetO-HSP90/hsp90*Δ strain in order to validate that the synergistic activity of geldanamycin with fluconazole against *C. albicans* biofilms was indeed due to Hsp90 inhibition. Biofilms of a wild-type strain of *C. albicans* had a fluconazole MIC_50_ of over 512 µg/mL (Figure 5B). Deletion of one allele of *HSP90* or replacing the promoter of the sole remaining *HSP90* allele with the tetracycline-repressible promoter had no impact on fluconazole resistance (Figure 5B). However, upon depletion of Hsp90 by doxycycline-mediated transcriptional repression in the *tetO-HSP90/hsp90*Δ strain, the fluconazole MIC_50_ was dramatically reduced to only 8 µg/mL, a >60-fold increase in fluconazole sensitivity (Figure 5B). Hence, both pharmacological and genetic evidence confirms that Hsp90 function is critical for azole resistance of *C. albicans* biofilms.

### The role of downstream effectors of Hsp90 in azole resistance of *C. albicans* biofilms

To further dissect the mechanism by which Hsp90 regulates azole resistance of *C. albicans* biofilms, we repeated the drug susceptibility assay with strains lacking specific Hsp90 client proteins. Under planktonic conditions both calcineurin and Mkc1 are important Hsp90 client proteins that regulate the maintenance of azole resistance [37], [38], [39]. Moreover, these client proteins have previously been shown to be important for azole resistance of *C. albicans* biofilms [30], [31]. We found that biofilms formed by strains lacking the catalytic subunit of calcineurin (*cna1Δ/cna1*Δ) or the terminal MAPK of the PKC cell wall integrity signalling pathway (*mkc1Δ/mkc1*Δ) had fluconazole MIC_50_ values of 32 µg/mL and 128 µg/mL, respectively; their fluconazole resistance levels were intermediate between the robust resistance of the wild-type parental strain and the sensitivity observed upon impairment of Hsp90 function (Figure 5B). The finding that compromise of calcineurin function does not confer as severe a reduction in biofilm fluconazole resistance as compromise of Hsp90 function is intriguing in light of the fact that under all planktonic conditions tested, inhibition of calcineurin phenocopies inhibition of Hsp90 in terms of azole resistance [37], [38], [49]. These results suggest that calcineurin and Mkc1 may be able to partially compensate for the loss of the other client during times of azole-induced stress in a biofilm environment. Alternatively, these findings could be explained by the existence of a novel downstream effector of Hsp90 important for azole resistance of *C. albicans* biofilms.

**Figure 6:**
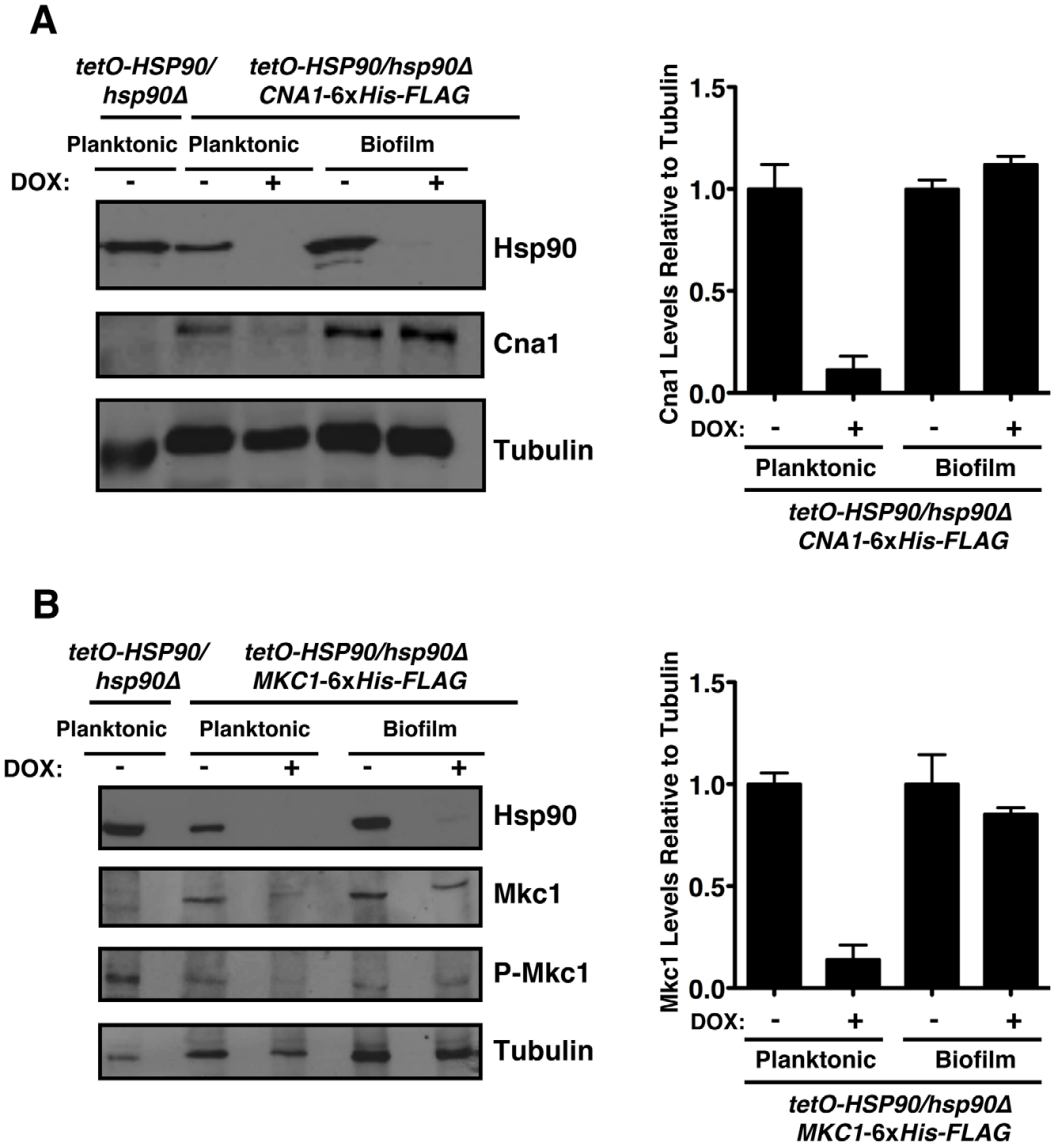
The Hsp90 client proteins Cna1 and Mkc1 exhibit reduced dependence on Hsp90 for stability under biofilm compared to planktonic conditions. (**A**) Genetic depletion of Hsp90 does not reduce calcineurin levels in biofilm conditions. The *tetO-HSP90/hsp90*Δ strain with one allele of *CNA1* C-terminally 6xHis-FLAG tagged was grown in planktonic or biofilm conditions with or without doxycycline (DOX, 20 µg/mL) for 48 hours. Total protein was resolved by SDS-PAGE and blots were hybridized with α-Hsp90, α-FLAG to monitor calcineurin levels, and α-tubulin as a loading control (left panel). Cna1 levels from two independent Western blots were quantified using ImageJ software (http://rsb.info.nih.gov/ij/index.html). The density of bands obtained for Cna1 was normalized relative to the density of bands for the corresponding tubulin loading control. Levels were subsequently normalized to the untreated control for the planktonic or biofilm state (right panel). (**B**) Depletion of Hsp90 does not deplete Mkc1 in biofilm conditions. The *tetO-HSP90/hsp90*Δ strain with one allele of *MKC1* C-terminally 6xHis-FLAG tagged was grown in planktonic or biofilm conditions with or without DOX for 48 hours. Total protein was resolved by SDS-PAGE and blots were hybridized with α-Hsp90, α-His_6_ to monitor Mkc1 levels, α-phospho-p44/42 to monitor dually phosphorylated Mkc1, and α-tubulin as a loading control (left panel). Mkc1 levels from two independent Western blots were quantified using ImageJ software. The density of bands for Mkc1 was normalized relative to the density of bands for the tubulin loading control. Levels were subsequently normalized to the untreated control for the planktonic or biofilm state (right panel).

To further investigate the mechanisms by which Hsp90 regulates azole resistance in biofilm conditions, we examined protein levels of the client proteins calcineurin and Mkc1 upon Hsp90 depletion. Strains were cultured in RPMI medium for both planktonic and biofilm growth. Biofilms were cultured on plastic under static conditions, as with our drug studies. We previously established that under planktonic conditions genetic reduction of Hsp90 levels leads to depletion of the catalytic subunit of calcineurin (Cna1) and Mkc1 [38], [39]. Here, the *tetO-HSP90/hsp90*Δ strain was grown in either planktonic or biofilm conditions in the presence or absence of 20 µg/mL doxycycline for 48 hours. Under both conditions, Hsp90 levels were dramatically reduced in the presence of doxycycline (Figure 6). To monitor calcineurin levels, we used a C-terminal 6xHis-FLAG epitope tag on Cna1 in the *tetO-HSP90/hsp90*Δ strain. In the tagged strains, Cna1 levels were comparable under planktonic and biofilm conditions in the absence of doxycycline (Figure 6A). Doxycycline-mediated reduction of Hsp90 levels led to an ∼90% reduction in Cna1 in planktonic conditions, however, Cna1 levels remained stable in biofilm conditions (Figure 6A). All strains had comparable amounts of protein loaded, as confirmed with a tubulin loading control. To monitor total Mkc1 levels, we used a C-terminal 6xHis-FLAG epitope tag on Mkc1 in the *tetO-HSP90/hsp90*Δ strain. Mkc1 levels were comparable in the tagged strains under planktonic and biofilm conditions in the absence of doxycycline (Figure 6B). As with Cna1, doxycycline-mediated reduction of Hsp90 levels led to ∼80% reduction in Mkc1 levels in planktonic conditions, however, Mkc1 levels remained stable in biofilm conditions (Figure 6B). We next addressed whether depletion of Hsp90 affected levels of activated, dually phosphorylated Mkc1. Mkc1 was activated in all strains in the absence of doxycycline. As with total Mkc1 levels, doxycycline-mediated reduction of Hsp90 led to a reduction in levels of activated Mkc1 in planktonic conditions, however, Mkc1 remained activated in biofilm conditions. Taken together, these results suggest that Hsp90 may play different roles in client protein regulation in these distinct cellular states, and also that these client proteins may have other means of maintaining stability in a biofilm environment.

### Hsp90 regulates matrix glucan levels in *C. albicans* biofilms

**Figure 7:**
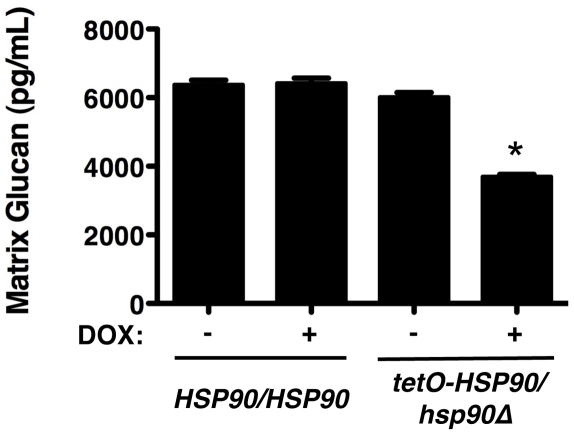
Depletion of Hsp90 reduces biofilm matrix glucan. Strains of *C. albicans* were cultured in 6-well polystyrene dishes for 48 hours with or without 20 µg/mL doxycycline (DOX). Matrix samples were collected and matrix β-1,3 glucan levels were meausured using a limulus lysate based assay. Asterisk indicates *P<*0.01 (ANOVA, Bonferroni's Multiple Comparison Test) compared to all other conditions.

Given our findings that Hsp90 client proteins remain stable in a biofilm, irrespective of Hsp90 levels, and that deletion of these client proteins does not phenocopy Hsp90 depletion in terms of biofilm azole resistance, we hypothesized that Hsp90 also regulates biofilm drug resistance through a mechanism independent of calcineurin and Mkc1 signalling. Recent studies established that glucan present in the biofilm matrix is critical for azole resistance due its capacity to sequester fluconazole, preventing it from reaching its intracellular target [29]. Consequently, we investigated whether Hsp90 affects glucan levels in the biofilm matrix. Biofilms were cultured on plastic in static conditions in the presence or absence of 20 µg/mL doxycycline for 48 hours, matrix material was harvested from biofilms with equivalent metabolic activity, and β-1,3 glucan levels were quantified. In the *tetO-HSP90/hsp90*Δ strain, the level of glucan in the biofilm matrix was ∼6,000 pg/mL in the absence of doxycycline (Figure 7). Transcriptional repression of *HSP90* with 20 µg/mL doxycycline led to reduced glucan levels of only ∼3,700 pg/mL (*P*<0.01, ANOVA, Bonferroni's Multiple Comparison Test, Figure 7). Doxycycline had no impact on matrix glucan levels of a wild-type strain lacking the *tetO* promoter, confirming that Hsp90 depletion leads to reduced glucan levels (Figure 7). The ∼40% reduction in matrix glucan upon Hsp90 depletion is likely to have made a major contribution to azole susceptibility, given that reduction of biofilm matrix glucan levels of ∼60% in an *FKS1/fks1*Δ mutant abrogates biofilm drug resistance [29]. These results provide the first link of Hsp90 to glucan production in *C. albicans* and mechanistic insight as to how Hsp90 regulates biofilm drug resistance.

### Hsp90 is required for *C. albicans* biofilm azole resistance in vivo

**Figure 8:**
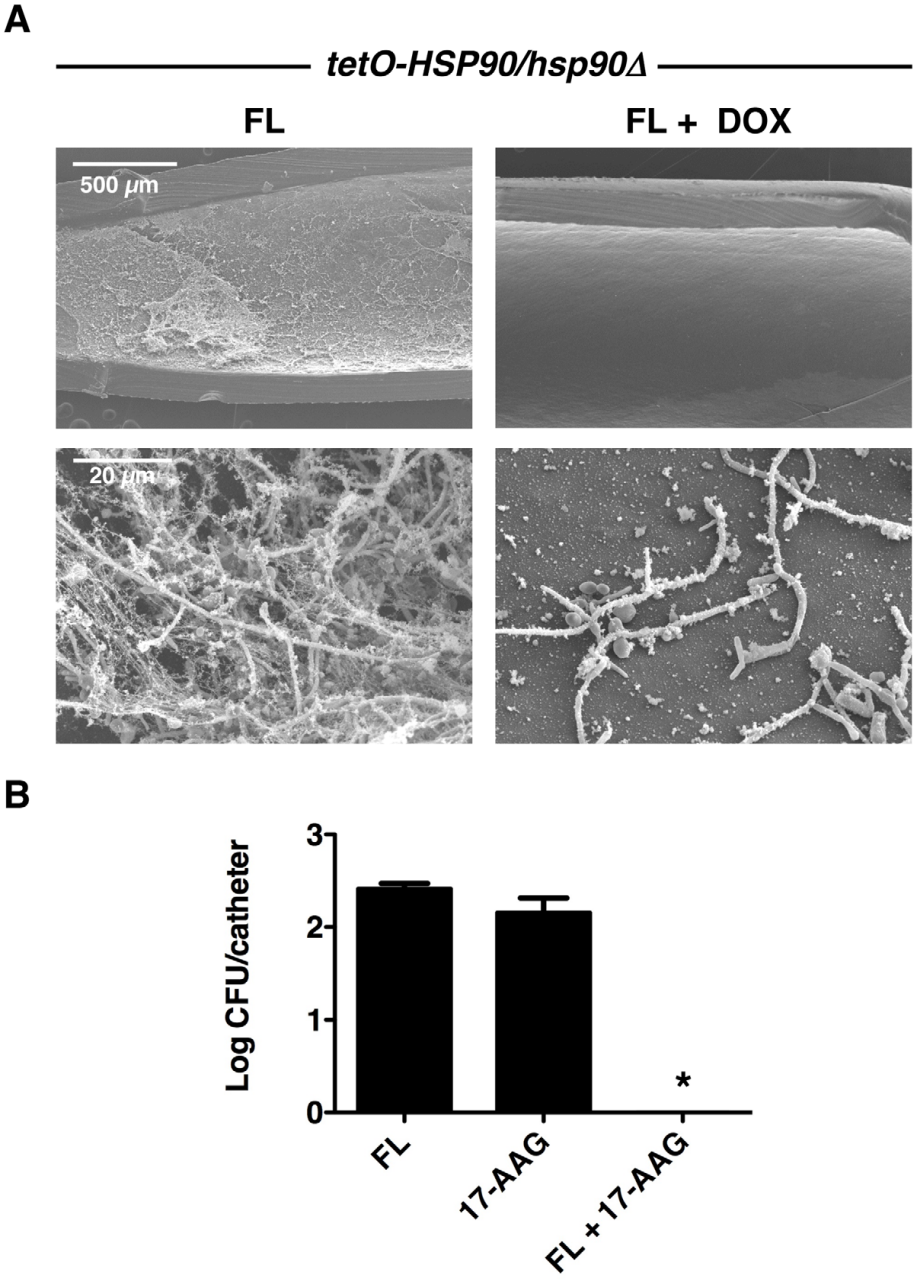
Compromise of Hsp90 function genetically or pharmacologically enhances the efficacy of fluconazole in vivo. (**A**) The *tetO*-*HSP90/hsp90*Δ strain was inoculated in rat venous catheters for 24 hours with or without 20 µg/mL doxycycline (DOX) followed by intraluminal azole treatment for an additional 24 hours. Following drug exposure, catheters were removed for visualization by scanning electron microscopy. The first column represents treatment with 250 µg/mL fluconazole (FL), followed by treatment with both 20 µg/mL DOX and 250 µg/mL FL. The top row represents 50 X magnification and the bottom row represents 1,000 X magnification. The combination of FL and DOX abrogates biofilms. (**B**) Biofilms were cultured as in A with 250 µg/mL FL, 100 µg/mL 17-AAG, or the combination of drugs. Serial dilutions of the catheter fluid were plated for viable fungal colony counts. Results are expressed as the mean colony forming unit (CFU) per catheter. The combination of FL and 17-AAG reduces fungal burden in the catheter compared to individual drug treatments (Asterisk indicates *P*<0.001, ANOVA, Bonferroni's Multiple Comparison Test).

Due to the robust synergy observed between Hsp90 inhibition and fluconazole in vitro, we sought to address whether synergy was also observed in vivo in the rat venous catheter model of *C. albicans* biofilm infection using the *tetO-HSP90/hsp90*Δ strain. Addition of fluconazole alone (250 µg/mL) after 24 hours of biofilm growth did not affect the biofilm formed by the *tetO-HSP90/hsp90*Δ strain (Figure 8A). Doxycycline was delivered during both the biofilm formation and drug treatment phases, and also had no major effect on the biofilm formed by the *tetO-HSP90/hsp90*Δ strain (Figure 2). However, the combination of fluconazole and doxycycline destroyed the biofilm as observed by scanning electron microscopy (Figure 8A). Thus, Hsp90 is required for the resistance of *C. albicans* biofilms to fluconazole in a mammalian host.

In order to further explore the therapeutic potential of targeting Hsp90 for *C. albicans* biofilm infections in vivo, we explored the efficacy of combining fluconazole with an Hsp90 inhibitor structurally related to geldanamycin and in clinical development as an anti-cancer agent, 17-(allylamino)-17-demethoxygeldanamycin (17-AAG). Central venous rat catheters were infected with *C. albicans* and biofilm formation proceeded over a 24-hour period. At this point, fluconazole alone (250 µg/mL), 17-AAG alone (100 µg/mL), or the drug combination was instilled and allowed to dwell in the catheter for an additional 24 hours. Serial dilutions of the catheter fluid were then plated in order to assess viable colony forming units. We found that the combined drug treatment significantly reduced fungal burden compared to the individual drug treatments alone (*P*<0.001, ANOVA, Bonferroni's Multiple Comparison Test, Figure 8B). In fact, catheters from the animals undergoing the combination therapy were completely sterile (Figure 8B). These experiments in a mammalian model provide compelling evidence that clinically relevant Hsp90 inhibitors may prove to be extremely valuable in combating *C. albicans* biofilm infections.

### Hsp90 is required for drug resistance of *A. fumigatus* biofilms

**Figure 9:**
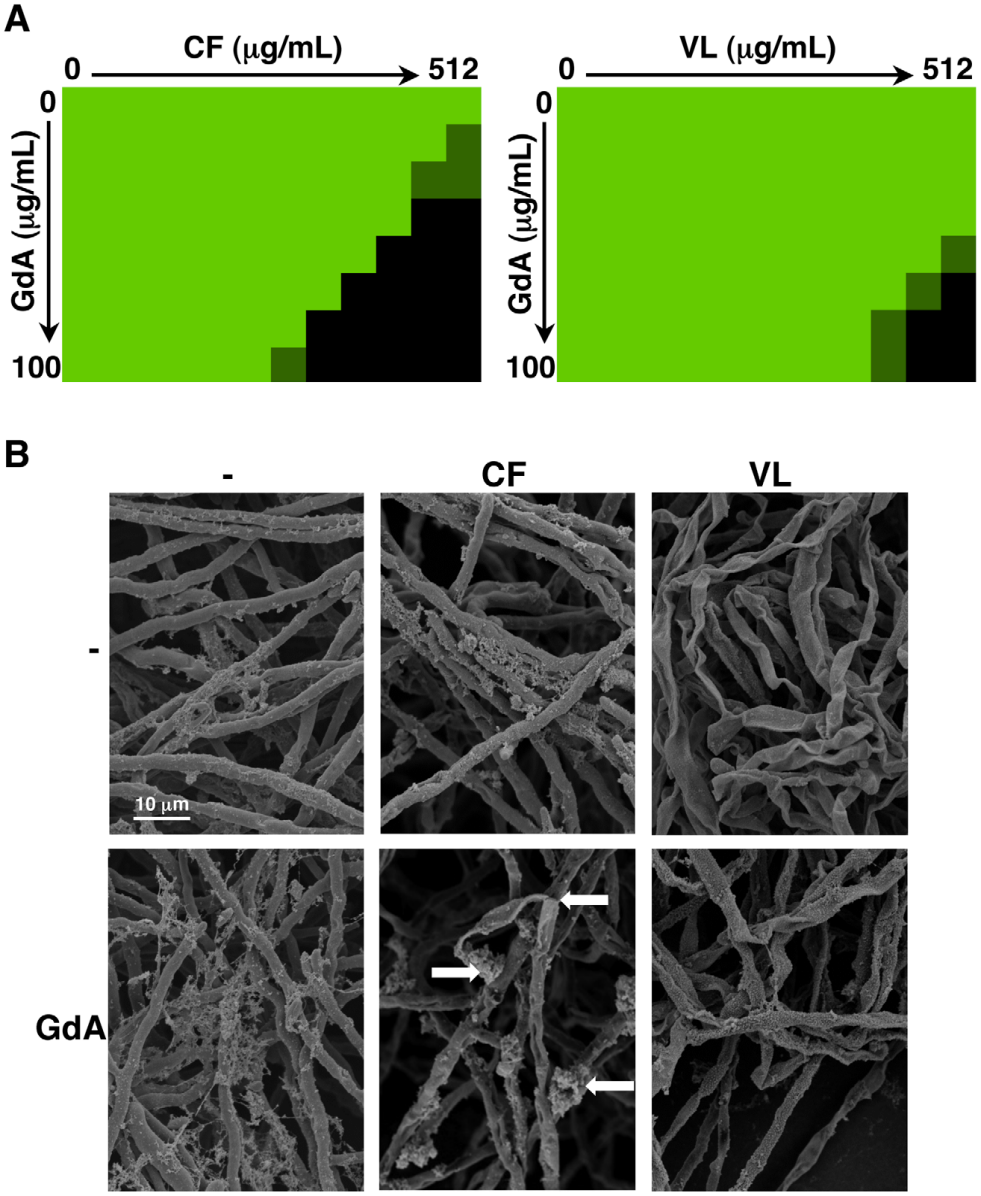
Pharmacological inhibition of Hsp90 enhances the efficacy of echinocandins and azoles against *A. fumigatus* biofilms and affects biofilm morphology. (**A**) *A. fumigatus* was grown in 96-well dishes in RPMI at 37°C. After 24 hours cells were washed with PBS to remove non-adherent cells and fresh medium was added with varying concentrations of the echinocandin caspofungin (CF), the azole voriconazole (VL), and the Hsp90 inhibitor geldanamycin (GdA) in a checkerboard format. Drug treatment was left on for 24 hours. Metabolic activity was measured as in Figure 1A. The FIC index was calculated as indicated in Table 2. Bright green represents growth above the MIC_50_, dull green represents growth at the MIC_50_, and black represents growth below the MIC_50_. (**B**) *A. fumigatus* cells were left untreated, or treated with 32 µg/mL CF or 256 µg/mL VL in the absence and presence of 50 µg/mL GdA for 24 hours. Following drug exposure, biofilms were fixed and imaged by scanning electron microscopy. Biofilms treated with antifungal show increased cellular damage in the presence of GdA. The white arrows indicate burst and broken hyphae in the biofilms treated with CF and GdA.

We previously established that Hsp90 inhibitors increase the efficacy of the echinocandins against *A. fumigatus* under standard culture conditions [34], motivating these studies to determine if Hsp90 inhibitors also affect drug resistance of *A. fumigatus* biofilms. After 24 hours of growth, *A. fumigatus* biofilms were subjected to a gradient of concentrations of the echinocandins caspofungin or micafungin, or the azoles voriconazole or fluconazole, in addition to a gradient of concentrations of the Hsp90 inhibitor geldanamycin in 96 well microtiter plates under static conditions. Metabolic activity was assessed using the XTT reduction assay after an additional 24 hours. The biofilms were completely resistant to all the antifungal drugs tested and geldanamycin individually, though the combination of geldanamycin with many of the antifungals was effective in reducing biofilm development. Geldanamycin displayed robust synergy with both caspofungin (Figure 9A) and micafungin (Figure S3A), with an FIC value of 0.375 for both drugs (Table 2). Geldanamycin also enhanced voriconazole activity (Figure 9A), with more potent effects observed when drugs were added to biofilms after only 8 hours of growth (Figure S3B). Geldanamycin did not enhance the efficacy of fluconazole under any conditions tested (data not shown). These patterns of drug synergy observed with *A. fumigatus* biofilms are consistent with those patterns observed with *Aspergillus* in planktonic conditions [37].

**Table 2:** Inhibition of Hsp90 has synergistic activity with echinocandins against wild-type *A. fumigatus* biofilms.

Antifungal concentration range (µg/mL)	GdA concentration range (µg/mL)	FIC index^a^
Micafungin, 64–512	25–100	0.375
Caspofungin, 128–512	12.5–100	0.375

a FIC index (MIC_50_ of drug A in combination)/(MIC_50_ of drug A alone) + (MIC_50_ of drug B in combination)/(MIC_50_ of drug B alone). A FIC of<0.5 is indicative of synergism.

Next, given Hsp90's role in regulating fungal morphogenesis we explored the impact of drug treatment on morphology of *A. fumigatus* biofilms. Scanning electron microscopy revealed striking architectural changes of *A. fumigatus* biofilms upon drug treatment. The control biofilms appeared robust and healthy, however, upon Hsp90 inhibition increased hyphal and matrix production was observed (Figure 9B). Treating biofilms with caspofungin alone resulted in minimal damage, however, the addition of both caspofungin and geldanamycin caused numerous burst and broken hyphae throughout the biofilm (Figure 9B). Finally, voriconazole treatment resulted in a flat ribbon-like morphology, and the addition of geldanamycin induced further cell damage (Figure 9B). Taken together, these results indicate that inhibition of Hsp90 induces changes in morphology of *A. fumigatus* biofilms, in addition to enhancing the efficacy of azoles and echinocandins against these otherwise recalcitrant cellular structures.

## Discussion

Our results establish a novel role for Hsp90 in dispersion and drug resistance of fungal biofilms, with profound therapeutic potential. Resistance of *C. albicans* biofilms to many antifungal drugs including the azoles, often necessitates surgical removal of the infected catheter or substrate demanding new therapeutic strategies. Here, we demonstrate that compromising the function of *C. albicans* Hsp90 blocks biofilm dispersal, potentially reducing their ability to serve as reservoirs for persistent infection (Figure 4). Further, we show that compromising Hsp90 function genetically or pharmacologically in *C. albicans* renders biofilms exquisitely susceptible to azoles, such that fluconazole is transformed from inefficacious to highly effective in destroying biofilms both in vitro (Figure 5 and Table 1) and in a mammalian model of infection (Figure 8). Finally, in *A. fumigatus* we found that compromising Hsp90 function dramatically improves the efficacy of antifungals (Figure 9). Thus, inhibition of Hsp90 enhances the efficacy of antifungals against biofilms formed by the two leading fungal pathogens of humans separated by ∼1 billion years of evolution, suggesting that this combinatorial therapeutic strategy could have a broad spectrum of activity against diverse fungal pathogens.

Hsp90 exerts pleiotropic effects on cellular circuitry in eukaryotes by stabilizing diverse regulators of cellular signalling [32], [33], [50]. Hsp90 regulates the temperature-dependent morphogenetic transition from yeast to filamentous growth in *C. albicans*, such that compromise of Hsp90 function by elevated temperature relieves Hsp90-mediated repression of Ras1-PKA signalling and induces filamentous growth [41]. While compromise of Hsp90 function could have impaired biofilm development by enhancing filamentous growth, we found negligible impact on biofilm development in vivo (Figure 2); in vitro, compromise of Hsp90 function did reduce biofilm maturation under static conditions with more severe effects under shaking conditions (Figures 1 and S1). Biofilms formed in the presence of Hsp90 inhibitor had a greater proportion of their total thickness occupied by filaments compared to yeast (Figure 3), suggesting that Hsp90's role in repressing the yeast to filament transition in planktonic cells [41] is conserved in the biofilm state. Consequently, we investigated the impact of compromising Hsp90 function on dispersion, a stage of the biofilm life cycle intimately coupled to morphogenetic transitions, with the majority of dispersed cells being in the yeast form [45], [46]. We found that compromising Hsp90 function dramatically reduces the dispersed cell population (Figure 4), consistent with previous findings with hyperfilamentous *C. albicans* mutants [45], [46]. Strikingly, the majority of cells that disperse from biofilms with reduced levels of Hsp90 are inviable (Figure 4), which likely reflects an enhanced dependence of this cell population on Hsp90. Given that the dispersed cell population is thought to be responsible for device-associated candidemia and the establishment of disseminated infection, inhibition of *C. albicans* Hsp90 function in individuals suffering from biofilm infections may assist in the prevention of the invasive forms of disease. In the broader sense, it is striking that depletion of Hsp90 blocks the production of yeast in *C. albicans* in planktonic conditions [41] as well as throughout the biofilm lifecycle, creating a constitutively filamentous program characteristic of the strictly filamentous lifestyle of the vast majority of fungi.

Hsp90 potentiates the emergence and maintenance of *C. albicans* drug resistance through multiple client proteins. A key mediator of Hsp90-dependent drug resistance is the protein phosphatase calcineurin [37], [38], [49]. In planktonic cells, Hsp90 stabilizes the catalytic subunit of calcineurin, Cna1, thereby enabling calcineurin-dependent cellular signalling required for survival of drug-induced cellular stress [38]. Hsp90 also regulates drug resistance by stabilizing the MAPK Mkc1, thereby enabling additional stress responses important for resistance [39]. In planktonic conditions, inhibition of calcineurin phenocopies inhibition of Hsp90 reducing drug resistance of diverse mutants, though deletion of *MKC1* has a less severe effect on resistance under specific conditions [37], [38], [39]. In biofilms, homozygous deletion of either *CNA1* or *MKC1* causes an intermediate increase in sensitivity to azoles compared to reduction of *HSP90* levels (Figure 5). Genetic depletion of Hsp90 reduces the fluconazole MIC_50_ from >512 µg/mL to 8 µg/mL, whereas deletion of *CNA1* reduces resistance to 32 µg/mL and deletion of *MKC1* reduces resistance only to 128 µg/mL (Figure 5). Thus, both calcineurin and Mkc1 have reduced impact on azole resistance of biofilms compared to Hsp90, suggesting differences in the Hsp90-dependent cellular circuitry between the biofilm and planktonic cellular states.

Hsp90 regulates circuitry required for fungal drug resistance largely by stabilizing key regulators of cellular signalling. In planktonic conditions, reduction of Hsp90 levels leads to depletion of both Cna1 and Mkc1 [38], [39]. In stark contrast, Cna1 and Mkc1 remain stable in biofilms, despite reduction of Hsp90 levels (Figure 6). In both planktonic and biofilm conditions, Hsp90 levels were reduced by doxycycline-mediated transcriptional repression in the *tetO-HSP90-hsp90*Δ strain and levels of Hsp90 were reduced sufficiently to abrogate drug resistance in both conditions. The reduced dependence of Cna1 and Mkc1 on Hsp90 in biofilms suggests that these proteins have altered stability in this cellular state. These Hsp90 client proteins may assume an alternate conformation in biofilms that is inherently more stable, or they may interact with other proteins or chaperones that confer increased stability and reduced dependence upon Hsp90. Consistent with the possibility of altered chaperone balance in biofilm cells, the Hsp70 family member *SSB1* is overexpressed six-fold in biofilms compared to their planktonic counterparts [51]. While it is possible that Hsp90 may still regulate Cna1 and Mkc1 function through a mechanism distinct from protein stability, we note that Mkc1 is still activated upon Hsp90 depletion in biofilms (Figure 6). Given Hsp90's high degree of connectivity in diverse signalling cascades, it could also affect biofilm drug resistance in a multitude of other ways, such as by regulating remodeling of the cell wall and cell membrane [27], [28], signalling cascades important for matrix production [29], [52], or the function of contact-dependent signalling molecules that initiate responses to surfaces [30]. Future studies will determine on a more global scale the impact of cellular state on Hsp90 client protein stability, and the complex circuitry by which Hsp90 regulates biofilm drug resistance.

Our results suggest that Hsp90 is a novel regulator of matrix glucan levels. For *C. albicans* the reduction in matrix glucan levels upon Hsp90 depletion provides a mechanism by which Hsp90 might govern biofilm azole resistance. *C. albicans* biofilms possess elevated cell wall β-1,3 glucan content compared to their planktonic counterparts [28], and matrix glucan sequesters fluconazole, preventing it from reaching its intracellular target [28], [29]. The ∼40% reduction in matrix glucan we observed upon Hsp90 depletion (Figure 7) likely contributes to reduced azole resistance, given that a reduction of matrix glucan levels of ∼60% in an *FKS1/fks1*Δ mutant abrogates biofilm drug resistance [29]. Hsp90 could regulate glucan levels by directly or indirectly affecting β-1,3 glucan synthase, Fks1, a protein important for the production of matrix glucan and for antifungal resistance [28], [29]. Alternatively, Hsp90 could regulate matrix production by directly or indirectly affecting Zap1, or its downstream targets Gca1 and Gca2, which play an important role in matrix production, likely through the hydrolytic release of β-glucan fragments from the environment [52]. We note that in *A. fumigatus*, inhibition of Hsp90 appears to increase matrix production (Figure 9), though glucan levels remain unknown. Future studies will dissect the molecular mechanisms by which Hsp90 regulates biofilm matrix production and if there is divergent circuitry between these fungal pathogens.

This work establishes that targeting Hsp90 may provide a powerful therapeutic strategy for biofilm infections caused by the leading fungal pathogens of humans. Compromising Hsp90 function genetically or pharmacologically reduces azole resistance of *C. albicans* biofilms both in vitro and in the rat venous catheter model of infection (Figures 5 and 8). Importantly, inhibition of Hsp90 with 17-AAG, an Hsp90 inhibitor that has advanced in clinical trials for the treatment of cancer [53], [54] and is synergistic with antifungals in planktonic conditions [34], transforms fluconazole from ineffective to highly efficacious in a mammalian model of biofilm infection (Figure 8). There may in fact be a multitude of benefits of inhibiting Hsp90 in the context of *C. albicans* biofilm infections given a recent report that treatment of in vitro *C. albicans* biofilms with voriconazole induces resistance to micafungin in an Hsp90-dependent manner [55]. The therapeutic potential of Hsp90 inhibitors against fungal biofilms extends beyond *C. albicans* to the most lethal mould, *A. fumigatus.* Pharmacological inhibition of Hsp90 enhances the efficacy of both azoles and echinocandins against *A. fumigatus* biofilms (Figure 9). The synergy between Hsp90 inhibitors and echinocandins is more pronounced than that with azoles, consistent with findings in the planktonic cellular state [34]. Thus, targeting Hsp90 may provide a much-needed strategy to enhance the efficacy of antifungal drugs against biofilms formed by diverse fungal pathogens.

Our results provide a new facet to the broader therapeutic paradigm of Hsp90 inhibitors in the treatment of infectious disease caused by fungi and other pathogenic eukaryotes. In addition to the profound effects on biofilm drug resistance and dispersal, compromising Hsp90 function enhances the efficacy of azoles and echinocandins against disseminated disease caused by the leading fungal pathogens of humans in invertebrate and mammalian models of infection [34], [38]. Beyond enhancing antifungal activity, Hsp90 also provides an attractive antifungal target on its own given that depletion of fungal Hsp90 results in complete clearance of a kidney fungal burden in a mouse model of disseminated candidiasis [41]. Hsp90 inhibitors also exhibit potent activity against malaria and *Trypanosoma* infections, thus extending their spectrum of activity to the protozoan parasites *Plasmodium falciparum* and *Trypanosoma evansi* [35], [36]. The development of Hsp90 as a therapeutic target for infectious disease may benefit from the plethora of structurally diverse Hsp90 inhibitors that have been developed, many of which are in advanced phase clinical trials for cancer treatment, with substantial promise due to the depletion of a myriad of oncoproteins upon inhibition of Hsp90 [56]. Given the importance of Hsp90 in chaperoning key regulators of cellular signalling in all eukaryotes, the challenge of advancing Hsp90 as a target for infectious disease lies in avoiding host toxicity issues. Indeed, although well tolerated in the mammalian host individually or in combination therapies [56], Hsp90 inhibitors have toxicity in the context of an acute disseminated fungal infection [34]. This toxicity may be due to Hsp90's role in regulating host immune and stress responses during infection. Toxicity was not observed in our studies of biofilm infections in the mammalian model, perhaps owing to both the localized infection and drug delivery, suggesting that this therapeutic strategy could rapidly translate from the laboratory bench to the patients' bedside. In the broader context, the challenge for further development of Hsp90 as a therapeutic target for infectious disease lies in developing pathogen-selective inhibitors or drugs that target pathogen-specific components of the Hsp90 circuitry governing drug resistance and virulence.

## Materials and Methods

### Ethics statement

All procedures were approved by the Institutional Animal Care and Use Committee (IACUC) at the University of Wisconsin according to the guidelines of the Animal Welfare Act, The Institute of Laboratory Animal Resources Guide for the Care and Use of Laboratory Animals, and Public Health Service Policy.

### Strains and culture conditions

Archives of *C. albicans* strains were maintained at −80°C in 25% glycerol. Strains were routinely maintained and grown in YPD liquid medium (1% yeast extract, 2% bactopeptone, 2% glucose) at 30°C. Strains used in this study are listed in Table S1. Strain construction is described in the *Supplemental Material*.

### Biofilm growth conditions

Multiple in vitro assays were used to assess *C. albicans* biofilm growth and antifungal drug susceptibility. In the first model, biofilms were developed in 96-well polystyrene plates, as previously described [28], [43]. Briefly, strains were grown overnight in YPD at 37°C. Subsequently, cultures were resuspended in RPMI medium buffered with HEPES or MOPS, in the presence or absence of doxycycline (631311, BD Biosciences) to a final concentration of 10^6^ cells/mL. An aliquot of 100 µl was added to each well of a 96-well flat-bottom plate, followed by incubation at 37°C. After 24 hours, the wells were gently washed twice with phosphate-buffered saline (PBS) to remove non-adherent cells, and fresh medium was added with or without a gradient of geldanamycin (ant-gl-5, Cedarlane). After 24 hours, non-adherent cells were washed away with PBS and biofilm cell metabolic activity was measured using the XTT reduction assay as previously described [28], [43]. Briefly, 90 µl of XTT (X4251, Sigma) at 1 mg/mL and 10 µl phenazine methosulfate (P9625, Sigma) at 320 µg/mL were added to each well, followed by incubation at 37°C for 2 hours. Absorbance of the supernatant transferred to a fresh plate was measured at 490 nm using an automated plate reader, and experiments were carried out in a minimum of 5 replicates for each strain.

In the second model, biofilms were developed on silicon elastomer (SE) surfaces as has been described previously [57]. *C. albicans* wild-type cells were grown overnight in YPD medium at 30°C and diluted to an optical density at 600 nm of 0.5 in RPMI medium. The suspension was added to a sterile 12-well plate containing bovine serum (B-9433, Sigma)-treated SE (Cardiovascular Instrument silicon sheets; PR72034-06N) and incubated at 37°C for 90 min at 150 rpm agitation for initial adhesion. The SE were washed with PBS, transferred to fresh plates containing either fresh RPMI medium in the absence of drug, or RPMI with 10 µg/mL geldanamycin or 20 µg/mL doxycycline. Plates were incubated at 37°C for 48 hours at 150 rpm agitation to allow biofilm formation, followed by visualization by microscopy or by monitoring biofilm growth by XTT reduction or dry weight, as previously described [31], [43].

### *C. albicans* biofilm dispersion

For obtaining cells dispersed from biofilms, *C. albicans* biofilms were cultured in a simple flow biofilm model, as described previously [47], [48]. Briefly, this model involves a controlled flow of fresh medium via Tygon tubing (Cole-Parmer, Vernon Hills, IL) into a 15 mL polypropylene conical tube (BD, Franklin, NJ) holding a SE strip. Medium flow is controlled at 1 mL/minute, by connecting the tubing to a peristaltic pump (Masterflex L/S Easy-Load II, Cole-Parmer). The whole apparatus is placed inside an incubator to facilitate biofilm development at 37°C. SE strips (1×9 cm_,_ Cardiovascular instrument Corp, Wakefield, MA), were sterilized by autoclaving and pre-treated for 24 hours with bovine serum. *C. albicans* was grown overnight at 30°C, washed, and diluted to an optical density at 600 nm of 0.5 in Yeast Nitrogen base (YNB) medium (BD Biosciences, San Jose, CA) with 50 mM glucose. The SE strips were incubated with the diluted *C. albicans* suspension at 37°C for 90 min at 100 rpm agitation for the initial adhesion of cells. Next, the strip was inserted into the conical tube and the peristaltic pump was turned on. At various time points during biofilm development, cells released from the biofilm in the flow-through were collected from the bottom of the conical tube. The dispersed cells were enumerated by a hemocytometer to obtain cell counts and there were no differences observed in the degree of clumping or morphological state of the dispersed cells, which were in the yeast form. Viability of the dispersed cells was assessed by plating and by colony counts on YPD agar.

### *C. albicans* in vitro biofilm drug susceptibility

Drug susceptibility assays were performed on biofilms formed in wells of 96-well plates. Fresh medium (RPMI/HEPES) and drugs were added to wells containing biofilms grown for 24 hours. Dilutions of fluconazole (Sequoia Research Products) were from 1000 µg/ml down to 0 with the following concentration steps in µg/ml: 1000, 500, 250, 125, 62.5, 31.25, 15.625, 7.8125, 3.90625, 1.953125, 0.9765625. FK506 (AG Scientific) gradients were from 75 µg/mL down to 0 with the following concentration steps in µg/ml: 75, 37.5, 18.75, 9.375, 4.6875, 2.3475, 1.171875. Geldanamycin gradients were from 100 µg/mL to 0 with the following concentration steps in µg/ml: 100, 50, 25, 12.5, 6.25, 3.125, 1.5625. Drug combinations were examined alone or in combination in a checkerboard format. After incubation at 37°C for 24 hours, biofilms were washed twice with PBS and metabolic activity was measured using the XTT assay, as described above. The drug concentration associated with 50% reduction in optical density compared to the drug-free control wells (MIC_50_) was determined. The fractional inhibitory concentration (FIC) was calculated as follows: [(MIC _50_ of drug A in combination)/(MIC _50_ of drug A alone)] + [(MIC_50_ of drug B in combination)/(MIC_50_ of drug B alone)]. Values of <0.5 indicates synergy, those of >0.5 but <2 indicate no interaction, and those of >2 show antagonism [28].

### *C. albicans* in vivo biofilm model

In order to evaluate biofilm formation in vivo, a rat central venous catheter infection model was employed [44]. Specific-pathogen-free Sprague-Dawley rats weighing ∼400 g were used (Harlan Sprague-Dawley, Indianapolis, IN). A heparinized (100 U/mL) polyethylene catheter was surgically inserted into the jugular vein and advanced 2 cm to a site above the right atrium. After the catheter was secured to the vein, the proximal end was tunneled subcutaneously to the midscapular space and externalized through the skin. The catheters were implanted 24 hours prior to inoculation with *C. albicans* to allow a conditioning period for deposition of host protein on the catheter surface. Infection was performed by intraluminal instillation of 500 µl of *C. albicans* (10^6^ cells/mL). After 6 hours, the catheters were flushed and maintained with heparinized 0.85% NaCl for 24 hours to allow for biofilm formation. While one end of the catheter is open to the venous blood, most of the fluid contents remain within the catheter unless pushed into the bloodstream with additional fluid from the external end. For drug treatment studies, fluconazole (250 µg/mL), 17-AAG (A-6880, LC Laboratories, 100 µg/mL), or saline was instilled and allowed to dwell in the catheter for an additional 24 hours [28]. For doxycycline studies, doxycycline (20 µg/mL) was delivered during both the biofilm formation and the drug treatment phases. At the end of the observation period, the animals were sacrificed and the catheters were removed. In order to quantify fungal biofilm formation in the catheter, the contents were drained to remove blood and non-adherent organisms. The distal 2 cm of catheter was cut from the entire catheter length and the segment was placed in 1 mL of 0.85% NaCl. Following sonication for 10 minutes (FS 14 water bath sonicator and 40-kHz transducer [Fisher Scientific]) and vigorous vortexing for 30 seconds, serial dilutions of the catheter fluid were plated on Sabouraud Dextrose Agar (SDA) for viable fungal colony counts. Results are expressed as the mean colony forming unit (CFU) per milliliter.

### *A. fumigatus* in vitro biofilm drug susceptibility

*Aspergillus fumigatus* Af293 was maintained on SDA slopes at 4°C. For conidial preparation Af293 was propagated on SAB agar for 72 hours and conidia harvested in PBS containing 0.025% (v/v) Tween 20 and quantified as previously described [58]. Commercially available voriconazole (Pfizer Pharmaceuticals, NY, USA), micafungin (Astellas Pharma Inc, Ibaraki, Japan) and caspofungin (Merck Sharp Dohme Ltd, NJ, USA) were used throughout this study. Each antifungal drug was prepared at stock concentrations of 10 mg/mL in sterile water and used within 24 hours of reconstitution.

Af293 conidial inoculum (1×10^5^ conidia/mL) was dispensed into flat bottomed 96-well microtitre plates and incubated for 8 or 24 hours at 37°C as previously described [58]. Biofilms were gently washed twice with PBS and each antifungal agent and geldanamycin were diluted to working concentrations in RPMI, which were tested either alone or in combination in a checkerboard format. Antifungal agent dilutions were from 512 µg/ml down to 0 with the following concentration steps in µg/ml: 512, 256, 128, 64, 32, 16, 8, 4, 2, 1, 0.5. Geldanamycin dilutions were from 100 µg/ml down to 0 with the following concentration steps in µg/ml: 100, 25, 12.5, 6.25, 3.125, 1.5625. The biofilms were then treated and processed as described for *C. albicans.*

### Confocal microscopy

Biofilms were stained with 25 µg/mL concanavalin A–Alexa Fluor 594 conjugate (C-11253; Molecular Probes, Eugene, OR) for 1 hour in the dark at 37°C. Confocal scanning laser microscopy (CSLM) was performed with a ZeissLSM 510 upright confocal microscope using a Zeiss Achroplan 40X, 0.8-W objective. Stained biofilms were observed using a HeNe1 laser with an excitation wavelength of 543 nm. The Zeiss LSM Image Browser v4.2 software was used to assemble images into side and depth views. Artificially coloured depth view images represent cell depth using a colour gradient, where cells closest to the SE are represented in blue and the cells farthest away are represented in red.

### Scanning electron microscopy

Biofilms formed in vitro were placed overnight in a fixative (4% formaldehyde v/v, 1% glutaraldehyde v/v in PBS), rinsed in 0.1 M phosphate buffer and air dried in desiccators. Notably, harsh dehydration steps were not performed to minimize the damage to the original biofilm structure. The samples were coated with gold/palladium (40%/60%) and observed under a scanning electron microscope (Leo 435 VP) in high vacuum mode at 15 kV. The images were assembled using Photoshop software (Adobe, Mountain View, CA.).

Catheter segments were processed for scanning electron microscopy as previously described [44]. Following overnight fixation (4% formaldehyde, 1% glutaraldehyde in PBS), catheter segments were washed with PBS and treated with osmium tetroxide (1% in PBS) for 30 minutes. Drying was accomplished using a series of alcohol washes followed by critical point drying. Catheter segments were mounted and gold coated. Images were obtained with a scanning electron microscope (JEOL JSM-6100) in the high-vacuum mode at 10 kV. The images were assembled using Adobe Photoshop 7.0.1.

### Immune blot analysis

For the protein stability assay, planktonic cultures were grown in RPMI buffered with MOPS and treated as described previously [39]. For biofilm cultures, *C. albicans* was grown overnight in YPD medium at 30°C and diluted to an optical density at 600 nm of 0.5 in RPMI medium. The suspension was added to a bovine serum (16190; Gibco)-treated sterile 6-well plate and incubated at 37°C for 90 minutes for initial adhesion. The plates were washed with PBS, and fresh RPMI medium was added with or without 20 µg/mL doxycycline. Plates were incubated at 37°C for 48 hours.

Cells were harvested by centrifugation and were washed with sterile water. Cell pellets were resuspended in lysis buffer containing 50 mM HEPES pH 7.4, 150 mM NaCl, 5 mM EDTA, 1% Triton X-100, 1 mM PMSF, and protease inhibitor cocktail (complete, EDTA-free tablet, Roche Diagnostics). Cells suspended in lysis buffer were mechanically disrupted by adding acid-washed glass beads and bead beating for 3 minutes. Protein concentrations were determined by Bradford analysis. Protein samples were mixed with one-sixth volume of 6X sample buffer containing 0.35 M Tris-HCl, 10% (w/v) SDS, 36% glycerol, 5% β-mercaptoethanol, and 0.012% bromophenol blue for SDS-PAGE. Samples were boiled for 5 minutes and then separated by SDS-PAGE using an 8% acrylamide gel. Proteins were electrotransferred to PVDF membranes (Bio-Rad Laboratories, Inc.) and blocked with 5% skimmed milk in phosphate buffered saline (PBS) with 0.1% tween. Blots were hybridized with antibodies against CaHsp90 (1∶10000), generously provided by Brian Larsen [59], FLAG (1∶10000, Sigma Aldrich Co.), His_6_ (1∶10, P5A11, generously provided by Elizabeth Wayner), phospho-p44/42 MAPK (Thr202/Tyr204) (1∶2000, Cell Signaling), or against alpha-tubulin (1∶1000; AbD Serotec, MCA78G).

### Biofilm matrix collection and matrix β-1,3 glucan measurements

Matrix β-1,3 glucan content was measured using a limulus lysate based assay, as previously described [28], [60]. Matrix was collected from *C. albicans* biofilms growing in the wells of 6-well polystyrene plates with or without 20 µg/mL doxycycline for 48 hours. The method for culturing biofilms was as described above for the immune blot analysis with the exception that all reagents were glucan-free. Biofilms were dislodged using a sterile spatula, washed with PBS, sonicated for 10 minutes, and centrifuged 3 times at 4500 x g for 20 minutes to separate cells from soluble matrix material [28], [61]. Samples were stored at -20°C and glucan concentrations were determined using the Glucatell (1,3)-Beta-D-Glucan Detection Reagent Kit (Associates of Cape Cod, MA) as per the manufacturer's directions.

### Accession numbers for genes and proteins mentioned in text (NCBI Entrez Gene ID number)

*C. albicans: PKC1* (3635298); *HSP90* (3637507); *CNA1* (3639406); *CNB1* (3636463); *MKC1* (3639710); *ERG11* (3641571); *FKS1* (3637073); *SSB1* (3642206); *GCA1* (3635124); *ZAP1* (3641162).

## Supporting Information

Figure S1 **The impact of Hsp90 depletion on** ***C. albicans*** **biofilm formation and maturation in multiple models**. (**A**) A wild-type strain of *C. albicans* and the *tetO-HSP90/hsp90*Δ strain were grown on silicon elastomer squares in RPMI at 37°C for 24 hours with or without 20 µg/mL doxycycline (DOX). Metabolic activity was measured as in Figure 1A. Treatment of wild-type biofilms with DOX did not alter biofilm growth, while Hsp90 depletion caused a moderate but significant reduction in biofilm growth (*P*<0.01, ANOVA, Bonferroni's Multiple Comparison Test). (**B**) Biofilms were grown as in part A, but growth was measured by dry weight. Treatment of wild-type biofilms with DOX did not alter biofilm growth, while Hsp90 depletion caused a moderate but significant reduction in biofilm growth (*P*<0.05). (C) The *tetO-HSP90/hsp90*Δ strain was grown with or without 20 µg/mL DOX in the overnight culture as well as during biofilm formation on plastic under static conditions. Metabolic activity was measured as in Figure 1A. Depletion of Hsp90 does not block biofilm formation. (**D**) Biofilms were cultured on plastic under shaking conditions with or without 20 µg/mL DOX. Treatment of wild-type biofilms with DOX did not alter biofilm growth. The *tetO-HSP90/hsp90*Δ strain showed impaired biofilm development (*P*<0.001), which was exacerbated in the presence of 20 µg/mL DOX. This is consistent with impaired *HSP90* induction in response to many conditions when driven by the non-native *tetO* promoter and the further transcriptional repression of *HSP90* with DOX [4].
(TIF)

Figure S2 **Treating** ***C. albicans*** **with doxycycline does not impair biofilm dispersal.** (**A**) A wild-type strain of *C. albicans* lacking the *tetO* promoter was cultured in the presence or absence of 20 µg/mL doxycycline (DOX). The number of dispersed cells released from biofilms was monitored over a 24 hour period. (**B**) The viability of dispersed cells from a wild-type *C. albicans* strain was determined by plating on YPD agar. DOX has no effect on biofilm dispersal or viability in a wild-type strain.
(TIF)

Figure S3 **Pharmacological inhibition of Hsp90 enhances the efficacy of echinocandins and azoles against** ***A. fumigatus*** **biofilms.** *A. fumigatus* was grown in 96-well microtiter plates in RPMI at 37°C. (**A**) After 24 hours cells were washed with PBS to remove non-adherent cells and fresh media was added with varying concentrations of the echinocandin micafungin (MF) in combination with the Hsp90 inhibitor geldanamycin (GdA) in a checkerboard format, and incubated with the biofilm for 24 hours. Metabolic activity was measured as in Figure 1A. The FIC index was calculated as indicated in Table 2. Bright green represents growth above the MIC_50_, dull green represents growth at the MIC_50_, and black represents growth below the MIC_50_. (**B**) After 8 hours cells were washed with PBS to remove non-adherent cells and fresh media was added with varying concentrations of the azole voriconazole (VL) in combination with GdA in a checkerboard format, and incubated with the biofilm for 24 hours. Metabolic activity was measured as in Figure 1A and data analyzed as in Figure S3A.
(TIF)

Table S1 ***C. albicans*** **strains used in this study.**
(DOC)

Text S1 **Supporting materials and methods.**
(DOC)
