# Supplementary figures and images for: Hsp90 Governs Dispersion and Drug Resistance of Fungal Biofilms
Source: PLoS Pathog. 2011 Sep 8;7(9):e1002257. doi: 10.1371/journal.ppat.1002257 (PMC3169563; doi:10.1371/journal.ppat.1002257)

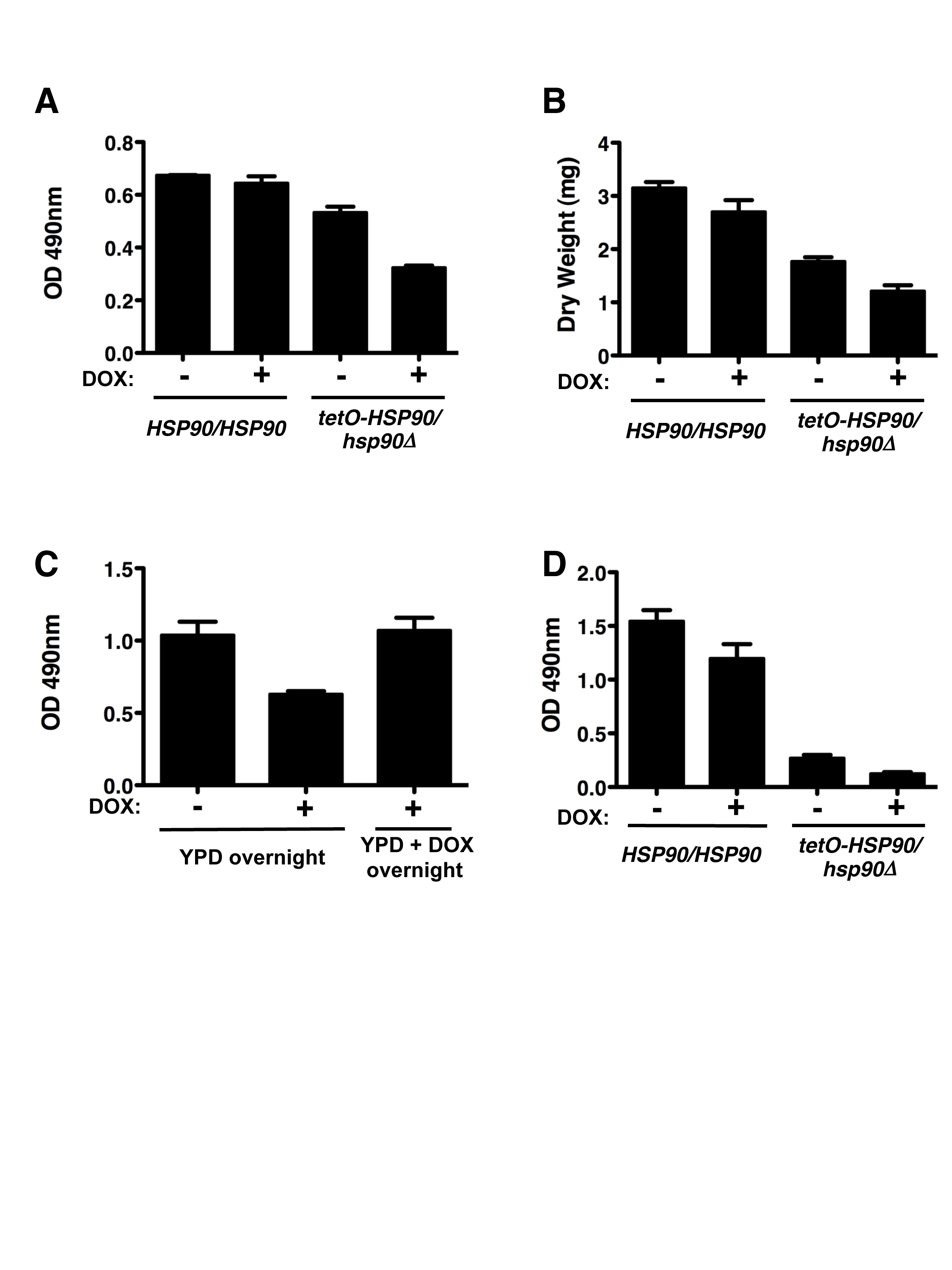

Supplement: Figure S1 — The impact of Hsp90 depletion on C. albicans biofilm formation and maturation in multiple models. (A) A wild-type strain of C. albicans and the tetO-HSP90/hsp90Δ strain were grown on silicon elastomer squares in RPMI at 37°C for 24 hours with or without 20 µg/mL doxycycline (DOX). Metabolic activity was measured as in Figure 1A. Treatment of wild-type biofilms with DOX did not alter biofilm growth, while Hsp90 depletion caused a moderate but significant reduction in biofilm growth (P<0.01, ANOVA, Bonferroni's Multiple Comparison Test). (B) Biofilms were grown as in part A, but growth was measured by dry weight. Treatment of wild-type biofilms with DOX did not alter biofilm growth, while Hsp90 depletion caused a moderate but significant reduction in biofilm growth (P<0.05). (C) The tetO-HSP90/hsp90Δ strain was grown with or without 20 µg/mL DOX in the overnight culture as well as during biofilm formation on plastic under static conditions. Metabolic activity was measured as in Figure 1A. Depletion of Hsp90 does not block biofilm formation. (D) Biofilms were cultured on plastic under shaking conditions with or without 20 µg/mL DOX. Treatment of wild-type biofilms with DOX did not alter biofilm growth. The tetO-HSP90/hsp90Δ strain showed impaired biofilm development (P<0.001), which was exacerbated in the presence of 20 µg/mL DOX. This is consistent with impaired HSP90 induction in response to many conditions when driven by the non-native tetO promoter and the further transcriptional repression of HSP90 with DOX [4]. (TIF) [file ppat.1002257.s001.tif]

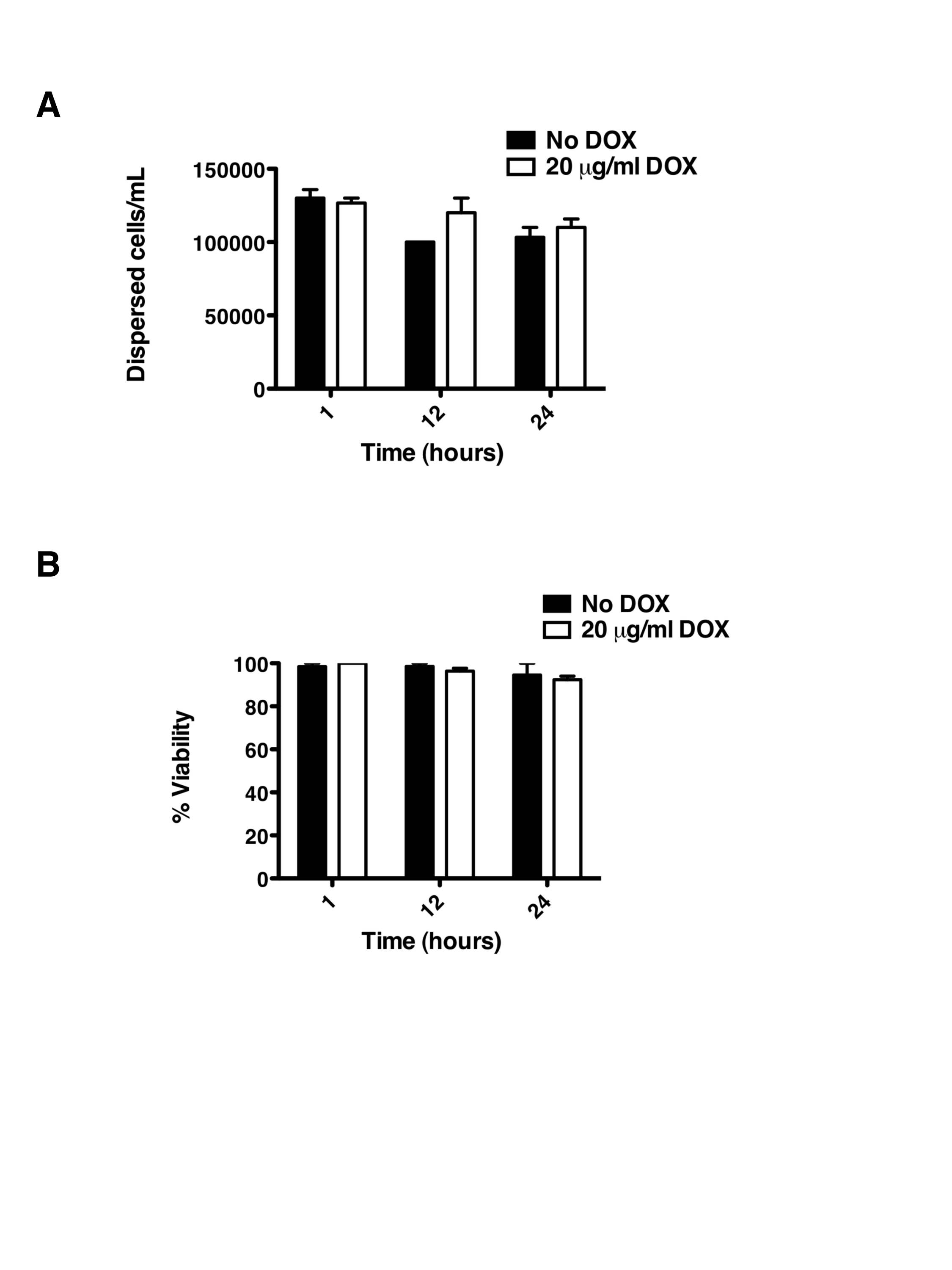

Supplement: Figure S2 — Treating C. albicans with doxycycline does not impair biofilm dispersal. (A) A wild-type strain of C. albicans lacking the tetO promoter was cultured in the presence or absence of 20 µg/mL doxycycline (DOX). The number of dispersed cells released from biofilms was monitored over a 24 hour period. (B) The viability of dispersed cells from a wild-type C. albicans strain was determined by plating on YPD agar. DOX has no effect on biofilm dispersal or viability in a wild-type strain. (TIF) [file ppat.1002257.s002.tif]

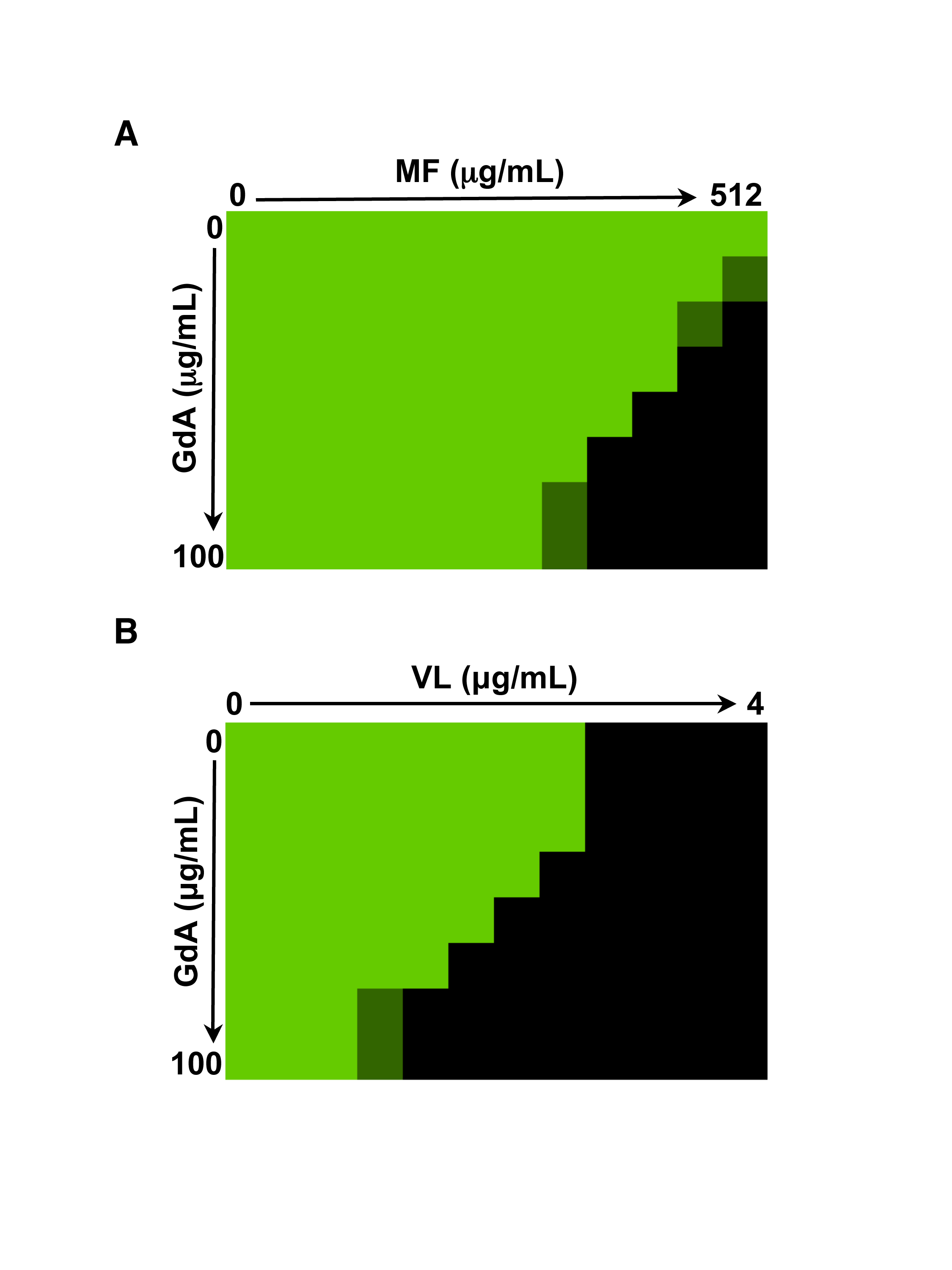

Supplement: Figure S3 — Pharmacological inhibition of Hsp90 enhances the efficacy of echinocandins and azoles against A. fumigatus biofilms. A. fumigatus was grown in 96-well microtiter plates in RPMI at 37°C. (A) After 24 hours cells were washed with PBS to remove non-adherent cells and fresh media was added with varying concentrations of the echinocandin micafungin (MF) in combination with the Hsp90 inhibitor geldanamycin (GdA) in a checkerboard format, and incubated with the biofilm for 24 hours. Metabolic activity was measured as in Figure 1A. The FIC index was calculated as indicated in Table 2. Bright green represents growth above the MIC50, dull green represents growth at the MIC50, and black represents growth below the MIC50. (B) After 8 hours cells were washed with PBS to remove non-adherent cells and fresh media was added with varying concentrations of the azole voriconazole (VL) in combination with GdA in a checkerboard format, and incubated with the biofilm for 24 hours. Metabolic activity was measured as in Figure 1A and data analyzed as in Figure S3A. (TIF) [file ppat.1002257.s003.tif]
