# Supplementary material for: Hsp90 Governs Dispersion and Drug Resistance of Fungal Biofilms
Source: PLoS Pathog. 2011 Sep 8;7(9):e1002257. doi: 10.1371/journal.ppat.1002257 (PMC3169563; doi:10.1371/journal.ppat.1002257)
Supplement: Table S1 — C. albicans strains used in this study. (DOC) [file ppat.1002257.s004.doc]

**Table S1. *C. albicans* strains used in this study.**

**______________________________________________________________________________**

Strain Name Genotype Source

**______________________________________________________________________________**

CaLC239 (SN95) *arg4∆/arg4∆ his1∆/his1∆* [2]

*URA3/ura3∆::imm434 IRO1/iro1∆::imm434*

CaLC367 As SN95, *HIS1/his1::TAR-FRT* [3]

*hsp90::CdHIS1/HSP90*

CaLC436 As SN95, *HIS1/his1::TAR-FRT* [3]

*hsp90::CdHIS1/FRT-tetO-HSP90*

CaLC592 As SN95, *HIS1/his1::TAR-FRT* This study

*hsp90::CdHIS1/FRT-tetO-HSP90*

*CNA1/CNA1-6xHIS-FLAG-FRT*

CaLC648 As SN95, *HIS1/his1::TAR-FRT* [4]

*hsp90::CdHIS1/FRT-tetO-HSP90*

*MKC1/MKC1-6xHIS-FLAG-FRT*

CaLC700 As SN95, *mkc1::FRT/mkc1::FRT* [4]

CaLC909 As SN95, *cna1::FRT/cna1::FRT* [1]

**______________________________________________________________________________**
