# Supplementary material for: Hsp90 Governs Dispersion and Drug Resistance of Fungal Biofilms
Source: PLoS Pathog. 2011 Sep 8;7(9):e1002257. doi: 10.1371/journal.ppat.1002257 (PMC3169563; doi:10.1371/journal.ppat.1002257)
Supplement: Text S1 — Supporting materials and methods. (DOC) [file ppat.1002257.s005.doc]

**Text S1**

**Supporting Materials and Methods**

***Strain Construction***

**CaLC592:** The plasmid pLC353 [1] was digested with KpnI and SacI to liberate the cassette to C-terminally 6xHis-FLAG tag *CNA1* and was transformed into CaLC436. Proper integration was verified by PCR with primers oLC342 (CaCNA1 + 1294F 5’-TTTAGTGAA GCTGAAATAGG – 3’) and oLC295 (CaHsp90 - 250R – ApaI 5’ – TTGCGGGCCCCTGTT ATAGGTAGTAATATGG – 3’) as well as oLC292 (pJK863down-F2 5’ – GCAAGCTT GATGGAAGTTCC - 3’) and oLC343 (5’ - CaCNA1 + 2355R AAGATCTGAGATCTTC TGCC - 3’). The *SAP2* promoter was induced to drive expression of FLP recombinase to excise the NAT marker cassette.

**Supplemental References**

1. Singh SD, Robbins N, Zaas AK, Schell WA, Perfect JR, et al. (2009) Hsp90 governs echinocandin resistance in the pathogenic yeast *Candida albicans* via calcineurin. PLoS Pathog 5: e1000532.

2. Noble SM, Johnson AD (2005) Strains and strategies for large-scale gene deletion studies of the diploid human fungal pathogen *Candida albicans*. Eukaryot Cell 4: 298-309.

3. Cowen LE, Singh SD, Köhler JR, Collins C, Zaas AK, et al. (2009) Harnessing Hsp90 function as a powerful, broadly effective therapeutic strategy for fungal infectious disease. Proc Natl Acad Sci USA 106: 2818-2823.

4. LaFayette SL, Collins C, Zaas AK, Schell WS, Betancourt-Quiroz M, et al. (2010) PKC signaling regulates drug resistance of the fungal pathogen *Candida albicans* via circuitry comprised of Mkc1, calcineurin, and Hsp90. PLoS Pathog 6: e1001069.
